# Anthropometric factors and the risk of ovarian cancer: A systematic review and meta‐analysis

**DOI:** 10.1002/cnr2.1618

**Published:** 2022-04-05

**Authors:** Bernadette Ellwanger, Susanne Schüler‐Toprak, Carmen Jochem, Michael F. Leitzmann, Hansjörg Baurecht

**Affiliations:** ^1^ Department of Epidemiology and Preventive Medicine University of Regensburg Regensburg Germany; ^2^ Department of Obstetrics and Gynecology University Medical Center Regensburg Regensburg Germany

**Keywords:** cancer epidemiology, meta‐analysis, obesity, ovarian neoplasm, overweight

## Abstract

**Background:**

Excess weight is convincingly associated with several cancers, but the association with ovarian cancer is insufficiently clarified, in particular regarding subgroups defined by menopausal status and ovarian cancer histologic type.

**Aims:**

We carried out a comprehensive systematic review and meta‐analysis of overweight and obesity in relation to ovarian cancer with focus on different subgroups.

**Methods and Results:**

We searched PubMed and Web of Science for relevant cohort and case–control studies published from inception to June 2021 in English language and using a clear definition of overweight and obesity. We combined maximally adjusted risk estimates using a random effects model. We analyzed data from 15 cohort and 26 case–control studies, including 28 471 ovarian cancer cases. The relative risk of ovarian cancer for overweight and obesity was 1.06 (95% confidence interval [CI] = 1.00–1.12) and 1.19 (95% CI = 1.11–1.28), respectively. Among premenopausal women, increased ovarian cancer risk was noted for overweight (RR 1.34; 95% CI = 1.03–1.75) and obesity (RR 1.51; 95% CI = 1.21–1.88). By comparison, among postmenopausal women no statistically significant association was found for overweight (RR 1.00; 95% CI 0.87–1.14) and obesity (RR1.03; 95% CI = 0.82–1.31). Increased risk was found for mucinous (RR 1.44; 95% CI = 1.03–2.01) and clear cell (RR 1.82; 95% CI = 1.11–2.99) ovarian cancer subtypes, but not for serous (RR1.12; 95% CI = 0.84–1.50;) and endometroid subtypes (RR1.24; 95% CI =0.96–1.60).

**Conclusions:**

Obesity is associated with increased ovarian cancer risk. That relation is largely due to a positive association between adiposity and ovarian cancer among premenopausal but not postmenopausal women and among cases with mucinous and clear cell but not serous or endometrioid histology.

## 1 INTRODUCTION

Ovarian cancer is the seventh most common malignancy among females worldwide, with an age standardized incidence rate (ASR) of 6.6 per 100 000 women. In 2018, ovarian cancer caused 184 799 deaths worldwide, accounting for 4.4% of the entire cancer‐related mortality among females.^1^, ^2^

A population‐based study in the UK estimated a 5‐year survival rate of 46% and indicates OC as the most lethal gynecological cancer.^3^ This very low survival rate can be explained by the often late detection of ovarian cancer in advanced stages.

Since local stage ovarian cancer has a 5‐year survival rate of 93%, improving prevention and detection methods should be a high research priority.^4^ For primary prevention, it is essential to understand the etiology of ovarian cancer and to identify risk factors as well as populations at high risk.^5^

The World Cancer Research Fund/American Institute for Cancer Research Report from 2018 stated the evidence of an association between obesity and ovarian cancer as probable but did not draw firm conclusions for specific subgroups.^6^

Two other meta‐analyses reported an association between excess weight and ovarian cancer,^7^, ^8^ but also provided information on particular subgroups.

To close this research gap, we performed a comprehensive systematic review and meta‐analysis of overweight and obesity in relation to ovarian cancer. Our study differs from previous reports by providing summary data on body mass index (BMI) categories as supported by the World Health Organization (WHO) in relation to ovarian cancer overall and according to subgroups defined by menopausal status, ovarian cancer histologic type, number of adjustment factors, self‐reported versus measured weight values, case–control versus cohort study design, and study geographic location.

## 2 MATERIAL AND METHODS

**FIGURE 1:**
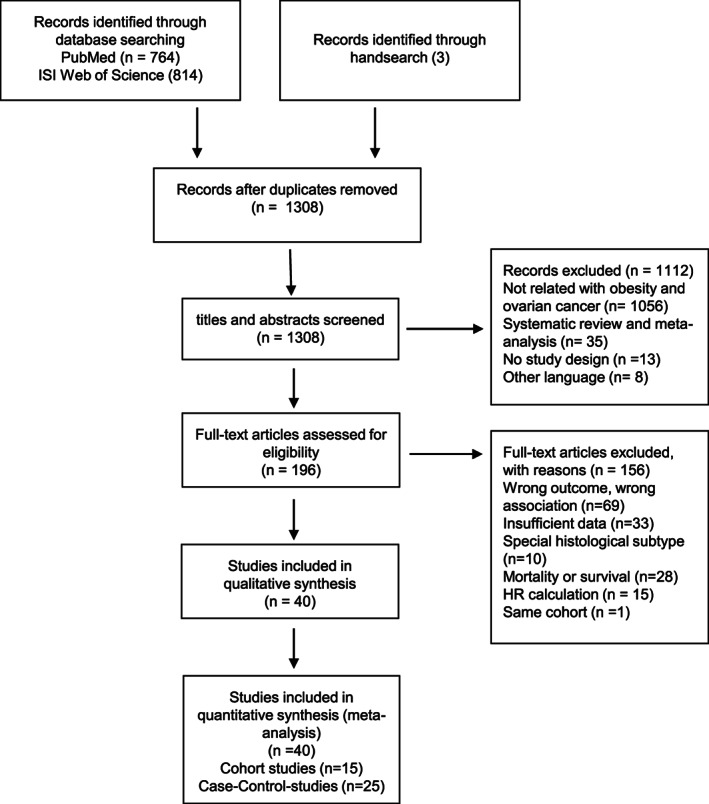
Preferred reporting items for systematic reviews and meta‐analyses (PRISMA) flow diagram depicting the process of study selection for meta‐analysis

The study was conducted following standard criteria for meta‐analyses according to the preferred reporting items for systematic reviews and meta‐analyses (PRISMA)^9^ (Figure 1). The PRISMA checklist is available in the supporting information (Resource 1). There is no pre‐registered study protocol.

### 2.1 Eligible criteria

All published prospective cohort studies and case–control studies addressing the link between ovarian cancer incidence and overweight or obesity were considered eligible. Gray literature, unpublished studies or studies in languages other than English were excluded. Further inclusion criteria were (1) clear definition of overweight or obesity by BMI and (2) availability of relative risk estimates or odds ratio together with 95% confidence intervals or standard errors. The focus lay upon epithelial ovarian cancer with specified histologic subtypes, but we also included studies that did not further define the type of ovarian tumor.

### 2.2 Search strategy

We carried out a systematic literature search in the scientific databases PubMed (764 results) and ISI Web of Science (814 results) to retrieve all relevant studies from inception to June 2021. Search terms are provided in the supporting information (Resource 2). In addition, we found three studies using hand search. BE screened the titles and abstracts. Following initial inclusion and exclusion criteria, BE read eligible articles and retrieved adequate studies. In case of uncertainty, a second researcher, HB, read the respective studies and inclusion decision was made by consensus. In addition, the reference lists of the included studies were hand‐searched to find any relevant studies with a similar research question.

### 2.3 Data extraction

The following information was extracted from each study: first author, publication year, study name and design, study population size and age range, geographic region, follow up duration, number of incident cases for cohort studies, number of cases and controls for case–control studies, histologic type (epithelial or total), case ascertainment, BMI measure (categorial, continuous), exposure measure (self‐reported, measured), BMI reference and exposure category, risk estimates (relative risk [RR] or odds ratio [OR]) with 95% confidence interval (CI) but not hazard ratios. The data extraction was done by one author (BE) and re‐examined by a second author (HB).

### 2.4 Statistical analysis

Risk estimates were referred to as relative risks (RR_*i*_). The logarithms of relative risks (log (RR_*i*_)) with their respective standard errors (*s*_*i*_ = *d*_*i*_/1.96) were calculated and *d*_*i*_ was defined as the maximum of (log(upper bound 95% confidence interval (CI) of RR_*i*_) − log(RR_*i*_)) and (log(RR_*i*_) − (log[lower bound 95% CI of RR_*i*_]). The logarithmic relative risks were weighted by $\omega_{i}=1/\left(s_{i}^{2}+\tau_{i}^{2}\right)$, using a random‐effects model, in which s_i_ describes the standard error of log(RR_*i*_) and $\tau_{i}^{2}$ the restricted maximum likelihood estimate (REML) of the between study variance allowing for heterogeneity.^10^

We used standard BMI categories of the WHO, which classify weight categories using the calculated BMI by weight and height as follows: underweight <18.5 kg/m^2^, normal weight 18.5–24.9 kg/m^2^, overweight 25.0–29.9 kg/m^2^ and obesity ≥30 kg/m^2^. For the eight studies in which the authors of the primary studies used non‐standard BMI categories, we selected the category that was closest to the WHO classification.

In the main analysis, we calculated summary RRs and 95% CI for overweight and obesity obtained from self‐reported or measured weight and height compared to normal weight as the reference category. Eight studies presented more detailed BMI categories, which we collapsed to match the WHO definition. For all studies, the most adjusted risk estimate was included in our meta‐analysis.

We further conducted a series of subgroup analyses with respect to BMI in early adulthood (ages 18–21 years), pre‐and post‐menopause and histologic subtypes of epithelial ovarian cancer (mucinous, endometroid, serous, clear cell, other).

Heterogeneity was assessed using the *Q*‐ and *I* ^2^‐statistic.^10^ Potential publication bias was assessed by funnel plot, Egger's^11^ and Begg's tests.^12^ In addition, we conducted sensitivity analyses including stratified analyses by study design, geographic region, excess weight definition and measurement, as well as outlier and influence diagnostics.^13^ Each study was evaluated for sources of bias using ROBINS‐I^14^ and the main findings of or meta‐analyses were assessed using GRADE.^15^

Results of our meta‐analyses are presented as risk estimates with corresponding 95% CI. *p* values < .05 were considered as statistically significant for the overall analysis. For subgroup analyses, we applied the false discovery rate (FDR) to correct for multiple testing.^16^ All statistical analyses were performed using the packages “robumeta”, “metafor” and “dplyr” in R (version 4.1.0).^17^

## 3 RESULTS

Our systematic literature search of electronic databases and hand‐searching resulted in 1581 potential studies (Figure 1). After removal of duplicates, 1308 studies remained for title and abstract screening, of which 196 were full text reviewed. Among these 196 publications, 69 were excluded due to inappropriate outcomes or exposure, 33 provided insufficient data, 10 referred to a histologic subtype not considered in the current analyses, 28 evaluated mortality or survival outcome and 15 investigated hazard ratios. One study^18^ used the same data as a more recent publication^19^ and was therefore excluded. Finally, a total of 40 studies was eligible and included in our meta‐analysis. Of those, 15 were cohort studies and 25 were case–control studies.

### 3.1 Study characteristics

The main characteristics of eligible studies are presented in Table S1. One publication provided combined results from two cohorts^20^ and another publication presented findings from three studies.^21^ One publication^19^ investigated two twin studies combined and presented results for younger and older subjects separately. We considered the latter two distinct data sets. We proceeded analogously with a study that presented results separately for African American and white populations.^22^

Thus, for our analyses, we pooled 42 independent studies in total, including 28 471 ovarian cancer cases and 3 499 022 study participants. A total of 31 studies investigated epithelial ovarian cancer and 11 studies did not specify a subtype (Table S1). Most studies (*N* = 32) used self‐reported anthropometric measures, while 10 studies relied on measured weight. Eighteen of the 42 studies were conducted in North America, 13 in Europe, seven in Asia and three in Australia (Table S2). The number of reported adjustment factors varied from 0 to 19 between studies. Most studies adjusted for age (20 studies), oral contraceptives (20 studies), parity (20 studies), family history of breast and ovarian Cancer (15 studies), region (11 studies) and hormone therapy (10 studies) Adjustment factors are listed in Table S3. All studies categorized BMI and estimated the risk of ovarian cancer for overweight and obesity compared to normal weight.

### 3.2 Overweight, obesity and ovarian cancer

**FIGURE 2:**
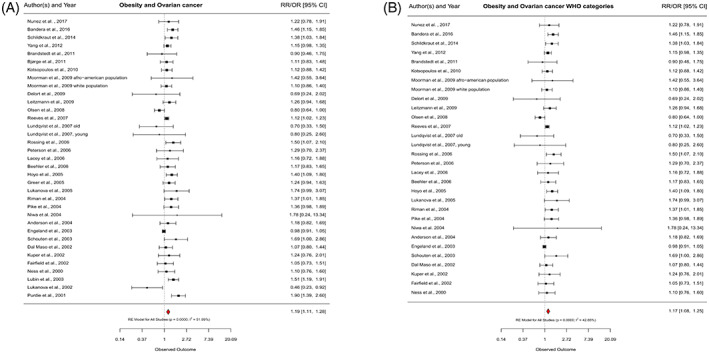
Association of overweight and ovarian cancer risk. Forest plots showing adjusted effect estimates and respective 95% confidence intervals (CI) for each study and overall effect using a random‐effects (RE) model. (A), all studies; (B), studies using the World Health Organization (WHO) definition for overweight (body mass index [BMI]; 25–29.9 kg/m^2^). RR, relative risk; OR, odds ratio; *p*, *p* value; *I* ^2^, heterogeneity between studies

**FIGURE 3:**
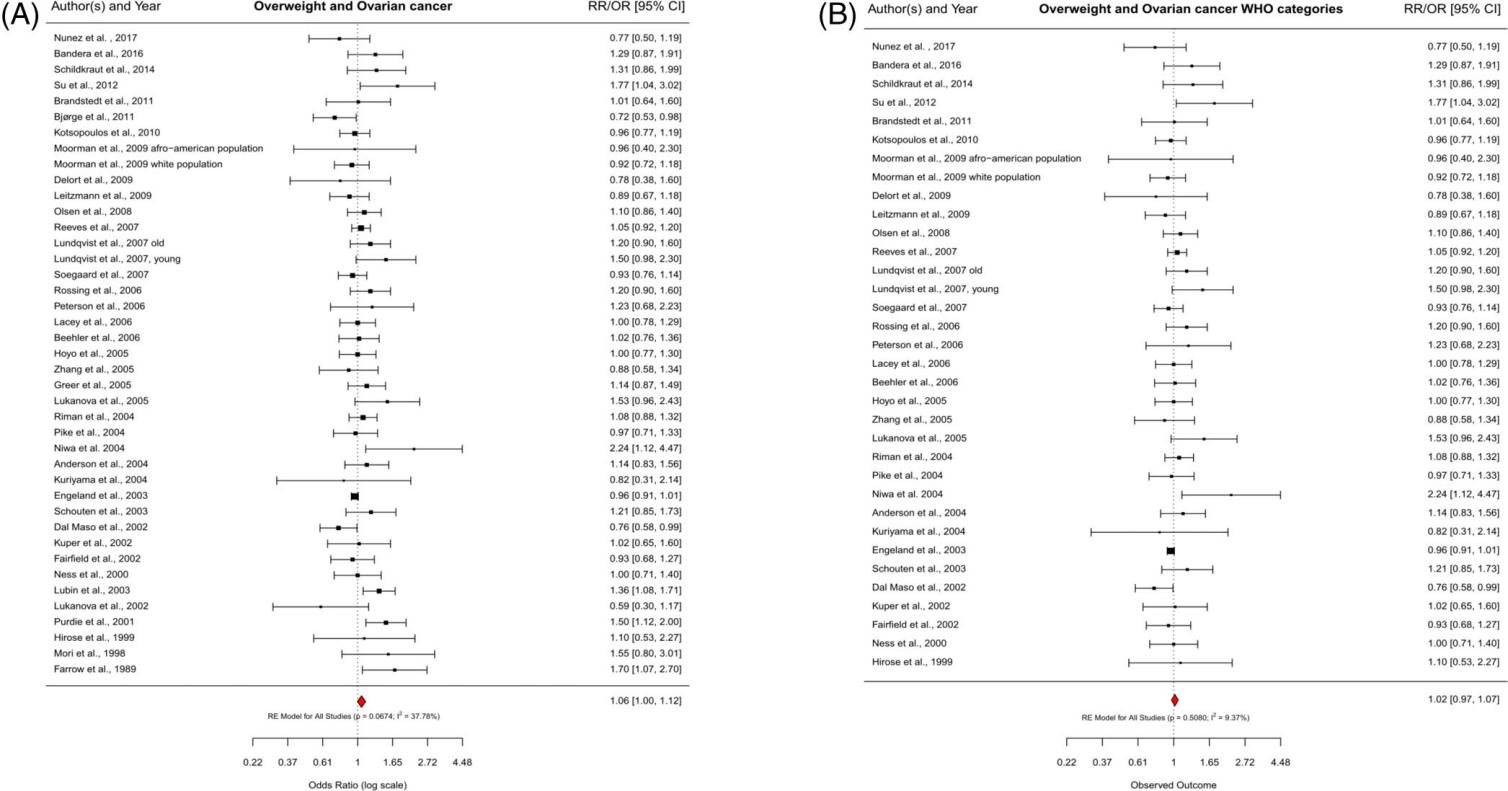
Association of obesity and ovarian cancer risk. Forest plots showing adjusted effect estimates and respective 95% confidence intervals (CI) for each study and overall effect using a random‐effects (RE) model. (A), all studies; (B), studies using the World Health Organization (WHO) definition for obesity (body mass index [BMI] ≥30 kg/m^2^). RR, relative risk; OR, odds ratio; *p*, *p* value; *I* ^2^, heterogeneity between studies

In the overall analysis, we observed a suggestively increased ovarian cancer risk for overweight compared to normal weight, including 41 of the 42 studies, with a RR of 1.06 (95% CI = 1.00–1.12) and minor between‐study heterogeneity (*I* ^2^ = 37.9%) (Figure 2A). For obesity, we pooled 35 studies and found a significantly increased risk for ovarian cancer (RR = 1.19; 95% CI = 1.11–1.28), with medium between‐study heterogeneity (*I* ^2^ = 52.0%) (Figure 3A). When we considered the 32 studies that used the WHO definition of BMI, above results were corroborated. While there was no clear association between overweight and ovarian cancer (RR = 1.02; 95% CI = 0.97–1.07) (Figure 2B), we again observed an increased ovarian cancer risk for obese versus normal weight women (RR = 1.17; 95% CI = 1.08–1.25) (Figure 3B). For both analyses, heterogeneity was reduced (WHO overweight: *I* ^2^ = 9.4%, *p*_heterogeneity_ = .2695; WHO obesity: *I* ^2^ = 42.8%, *p*_heterogeneity_ = .0162).

We next performed stratified analyses to detect potential causes of heterogeneity.

**FIGURE 4:**
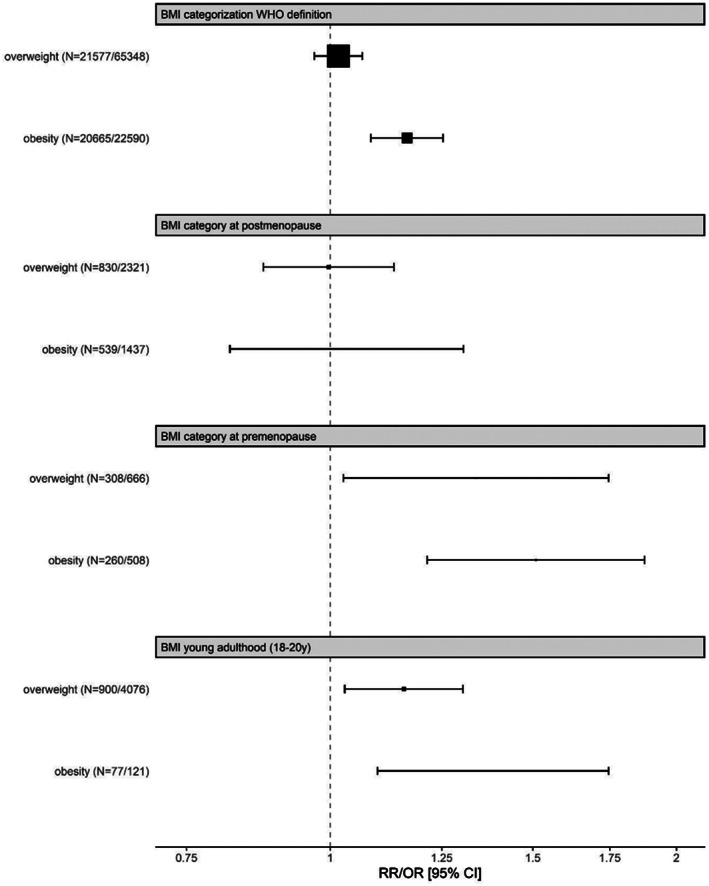
Association of overweight and obesity and ovarian cancer risk for different subgroup‐analyses: body mass index (BMI) World Health Organization (WHO) categories, BMI at pre‐ and post‐menopause and BMI at young adulthood. RR, relative risk; OR, odds ratio

When looking at adiposity during different periods in life, ovarian cancer risk was increased for both overweight (RR = 1.16; 95% CI = 1.03–1.30) and obesity (RR = 1.39; 95% CI = 1.10–1.75) in young adults as well as in pre‐menopausal women (RR for overweight = 1.34; 95% CI = 1.03–1.75; RR for obesity = 1.51; 95% CI = 1.21–1.88), with low to moderate heterogeneity (Figure 4, Figures S1 and S2, Table S2).By comparison, we did not observe significant ovarian cancer risk for postmenopausal excess weight categories (RR for overweight = 1.00; 95% CI = 0.87–1.14; RR for obesity = 1.03; 95% CI = 0.82–1.31). Heterogeneity was low to modest (*I* ^2^ = 0.00%, *p*_heterogeneity_ = .0165); *I* ^2^ = 54.6%, *p*_heterogeneity_ = .0165) for the analysis of postmenopausal overweight and obesity and ovarian cancer risk (Figure 4, Figure S3, Table S2).

When analyzing histologic subtypes of epithelial ovarian cancer, we found a statistically significant increased risk for obesity and mucinous (RR = 1.44, 95% CI = 1.03–2.01), clear cell (RR = 1.82, 95% CI = 1.11–2.99) and undifferentiated (RR = 1.57, 95% CI = 1.22–2.03) ovarian cancer subtypes (Table S2). No statistically significant relation was observed for obesity and serous (RR = 1.12; 95% CI = 0.84–1.16) or endometroid ovarian cancer subtype (RR = 1.24; 95% CI = 0.96–1.60) (Table S2).

We carried out a series of additional stratified analyses to further explore potential sources of heterogeneity. We observed greater heterogeneity between risk estimates for ovarian cancer in case–control studies compared to cohort studies for both overweight and obesity. While both study types revealed obesity as a statistically significant risk factor for ovarian cancer (case–control studies: RR = 1.25; 95% CI = 1.12–1.39; cohort studies: RR = 1.10; 95% CI = 1.02–1.19), no increased risk was observed for overweight (case–control studies: RR = 1.08; 95% CI = 0.99–1.17; cohort studies: RR = 1.02; 95% CI = 0.95–1.09) (Table S2).

When considering different geographic regions, we found more pronounced ovarian cancer risk estimates in Asia along with low heterogeneity for both overweight and obesity, while studies in all other geographic regions revealed weaker risk estimates with moderate to strong heterogeneity. Interestingly, no statistically significantly increased ovarian cancer risk was observed for obese women in Europe and Australia, while in the 18 North American studies risk was significantly increased with low heterogeneity (Table S2).

Statistically significant increased ovarian cancer risk was observed for overweight when adjusted for parity or smoking status and for obesity when adjusted for age at recruitment, age at menarche, parity, contraceptives, menopausal hormone therapy, tubal ligation, number of live birth or family history of breast and/or ovarian cancer (Table S2).

We found divergent results between studies that used BMI based on measured weight and height versus those that used BMI based on self‐reported weight and height. While the 32 studies with self‐reported values showed a statistically significant positive relation for both overweight and obesity with ovarian cancer with high heterogeneity, no significant association was observed for the 10 studies with measured values (Table S2).

### 3.3 Sensitivity analyses, influence diagnostics and publication bias

For both overweight and obesity, funnel plots did not show strong asymmetry by visual inspection (Figure S4a,c), corroborated by Egger's (*p*_obesity_ = .8041; *p*_overweight_ = .1133) and Begg's test (*p*_obesity_ = .3305; *p*_overweight_ = .1963), indicating no publication bias. Accordingly, the trim and fill method also did not show strong deviation from symmetry (Figure S4b,d).

Influence diagnostics by omitting one study at a time did not reveal substantially altered results, with summary risk estimates ranging from RR = 1.04 (95% CI = 0.98–1.10) to RR 1.07 (95%CI = 1.00–1.14) for overweight and RR = 1.17 (95% CI = 1.09–1.26) to RR = 1.22 (95% CI = 1.13–1.31) for obesity (Tables S4 and S5). Accordingly, influence diagnostic for subgroup analyses also showed no substantial impact on the results (Tables S6 and S7). When evaluating each study for potential bias, we found any studies with moderate information respectively recall bias based on self‐report of weight and height (Table S8). Assessment of our main findings using GRADE revealed low evidence quality since all included studies were observational studies (Table S9).

## 4 DISCUSSION

Our meta‐analysis updates the current knowledge of the relation of overweight and obesity to ovarian cancer, including over 15 000 additional ovarian cancer cases and 14 additional studies compared to previous reports.^7^, ^8^ In addition, we present novel information on overweight and obesity in relation to ovarian cancer risk according to histologic ovarian cancer subtypes and adiposity during different periods of life. While overweight women have only a suggestively increased risk of 6% for ovarian cancer, we found a strong increase of risk of 19% for obese women. For obesity this is in line with the results of the International Agency for Research on Cancer (IARC)^23^ and a recent umbrella review on gynecological cancers.^24^ Also, a Mendelian randomization study suggested a positive association between obesity and ovarian cancer.^25^ By comparison, a dose–response meta‐analysis did not find strong evidence for an association of BMI and ovarian cancer.^26^

Considering different time points in a woman's life, our results indicate that overweight and obesity increases the risk of ovarian cancer particularly in premenopausal women. In line with our findings, a positive association of BMI and premenopausal ovarian cancer was found. by a recent Mendelian randomization study in premenopausal *BRCA1* and *BRCA2* mutation carriers.^27^ By comparison, overweight and obesity appear to have little impact on ovarian cancer risk in postmenopausal women. This suggests that early onset adiposity leading to long‐lasting oncogenic effects on ovarian function results in malignant transformation in this organ.

**FIGURE 5:**
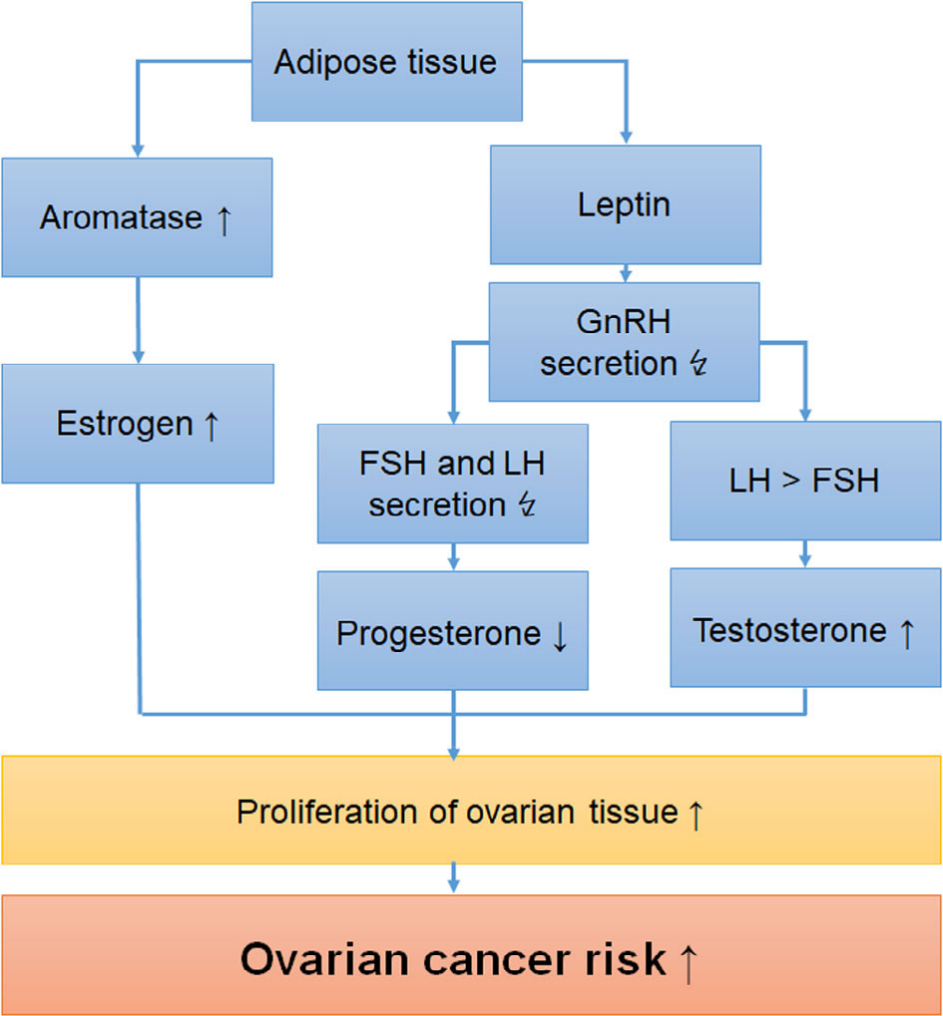
Biological mechanisms by which excess adiposity may increase the risk of ovarian cancer

The biological mechanisms by which greater body weight increases the risk for ovarian cancer are yet not fully understood. Several hypotheses, including endogenous hormones and growth factors (IGF‐1), could explain the association (Figure 5).^28^

In obese women secretion of gonadotropin‐releasing hormone (GnRH) is impaired. Leptin, which is secreted by adipose tissue, affects GnRH pulse neurons through gamma‐aminobutyric acid (GABA) and kisspeptin.^29^ Moreover, elevated serum levels of estrogen and testosterone, as observed in women with adiposity, yield a negative feedback mechanism resulting in decreased secretion of GnRH. GnRH controls the pituitary secretion of gonadotropins. This decapeptide and its receptors are found in the ovarian surface epithelium as well as in ovarian cancers.^30^ However, to date little is known about the physiologic and pathophysiologic roles of GnRH in the human ovary and a possible effect of impaired GnRH secretion on ovarian cancer risk.

As a consequence of disturbed GnRH secretion and high serum levels of estrogen and testosterone, obese women show low levels of follicle‐stimulating hormone (FSH) and luteinizing hormone (LH).^29^ As pulsatile GnRH frequency is impaired, LH release dominates FSH, resulting in elevated serum androgen levels. FSH and LH are secreted in the pituitary gland. Their receptors located in the ovary regulates the secretion of ovarian steroids. Expression of these receptors decreases during the dedifferentiation process. Receptors for gonadotropins have been found in cystadenomas, which are considered precursor lesions of certain types of ovarian cancers, in 80% of cases.^31^

Estrogen has strong proliferative effects on ovarian cancer cells.^32^ Aromatase, which is located in adipocytes, but is also found in ovarian cancers, is responsible for conversion of testosterone to estradiol, leading to high estrogen levels in obese women. In our study, we found an increased risk of ovarian cancer especially for young obese women. This can be explained by the persistent influence of estrogens on the ovarian surface epithelium resulting in induction of proliferation.^33^, ^34^ The Women's Health Initiative (WHI) Study found that the association between circulating levels of estrone and ovarian cancer varies by histologic subtype. While a statistically significant positive association with estrone levels was observed in non‐serous ovarian cancers, no relation was found in serous tumors.^35^

In line with this, we found an increased risk between obesity and mucinous and clear cell ovarian cancers, although the precision of the risk estimates in our meta‐analysis was low due to the small number of cases. The luteal phase is impaired in overweight women, which can be attributed to a decreased pituitary LH pulse leading to a lower release of LH. The compromised luteal phase results in depressed serum progesterone levels.^29^ High doses of progesterone decreased growth of ovarian cancer cells and induced apoptosis in vitro.^36^ In our study, we observed an increased risk for mucinous ovarian cancer in obese women. In line with this, Gomora et al. found a decreased progesterone receptor expression in mucinous subtype of ovarian cancers.^37^ As low serum progesterone levels are exhibited in women with adiposity, ovarian cancer risk reducing progesterone mechanisms cannot take place. This supports our data showing an increased ovarian cancer risk for obese women.

Women with adiposity exhibit elevated serum androgen levels. High expression of androgen receptors has been observed in ovarian cancer cells.^38^ Even during the process of dedifferentiation of ovarian cancers, the expression of androgen receptors was found to be conserved. Especially the putative precursor locations tubal fimbriae and ovarian surface epithelium highly express the androgen receptors. Growth stimulation effects of androgens on ovarian surface epithelial cells have been shown.^39^ These effects underscore our data showing a significantly increased risk of ovarian cancer in obese women, who are known to have elevated serum androgen levels.

According to the androgen/progesterone theory, high levels of androgens affecting ovarian epithelial cells increase ovarian cancer risk. An increase of progesterone stimulation on the other hand leads to decreased risk of ovarian cancer.^40^

This theory is strongly supported by the strong known protective effects of parity on ovarian cancer risk as high levels of progesterone are secreted during pregnancies. Another factor supporting this hypothesis is the observation of decreased ovarian cancer risk after intake of progestin‐only oral contraceptives.

Underlining the androgen/progesterone theory several studies showed an increase of ovarian cancer risk for women exhibiting symptoms like acne, hirsutism and increased waist‐to‐hip‐ratio, all signs for increased androgen levels.^34^, ^41^

Our data showing an increased ovarian cancer risk in young and premenopausal women is strongly supported by the androgen/progesterone theory.

To date, EOC has largely been considered a single disease. However, ovarian cancer is increasingly recognized as a collection of up to five distinct entities, including high‐grade serous, low grade serous, endometroid, clear cell and mucinous carcinomas.^42^ We found a robust association of obesity only with the mucinous and clear cell subtypes. For all other subtypes, the body of evidence is weak due to small numbers of studies (2 ≤ *n* ≤ 6) and cases (57 ≤ *n* ≤ 1433). Interestingly, we did not find an association of obesity with the endometroid subtype, although we analyzed a substantial number of studies (*n* = 6) comprising 306 ovarian cancer cases. Thus, our findings do not support the notion that the pathology of endometroid ovarian cancer is similar to that of endometrial cancer,^43^ for which obesity is a well‐known risk factor. Further studies are needed to clarify the role of different ovarian cancer histologic types in this context.

### 4.1 Strength and limitations

#### 4.1.1 Our study has some limitations

The majority of underlying studies used self‐reported rather than objectively measured weight and height. The 10 studies that used measured weight showed a significantly weaker risk estimate especially for the obese category, which indicates some degree of measurement error or information bias. Evidence shows an overestimation of height and an underestimation of weight when it comes to self‐reported information, especially in women of younger age groups.^44^, ^45^ However, potential discrepancies are still considered at a level that allows the use of self‐reported weight as a proxy of true values in both clinical and research evaluations. An additional shortcoming of our study is the Significant heterogeneity encountered, of which a large proportion was explained by study specific differences in the definition of BMI and the timing of BMI measurement during life. Finally, sufficient data to assess potential effect modification by hormone replacement therapy were not available.

Our study also has some remarkable strengths. It pooled data from over 40 studies, representing a comprehensive meta‐analysis on overweight and obesity in relation to the risk of ovarian cancer. None of the studies suffered from significant sources of bias. Its large sample size provided substantial statistical power and allowed us to perform numerous informative sub‐analyses. We combined evidence based on different time points in a woman's life and presented subgroup analyses on the different histologic subtypes of epithelial ovarian cancer. Although our results are based solely on observational studies, the quality of evidence did not have to be devalued by any factors affecting the quality.

In conclusion, our meta‐analysis provides evidence for an increased risk of developing ovarian cancer with a strongly elevated BMI, in particular during the fertile phase of life.

As the prevalence of obesity is increasing in developed as well as in developing countries, our findings potentially have an important impact on public health, which will be confronted with finding preventive measures for the rising problems that come with obesity in the future.

## CONFLICT OF INTEREST

The authors have stated explicitly that there are no conflicts of interest in connection with this article.

## AUTHOR CONTRIBUTIONS

**Bernadette Ellwanger:** Conceptualization (equal); data curation (equal); formal analysis (equal); methodology (equal); project administration (equal); validation (equal); visualization (equal); writing – original draft (equal). **Susanne Schüler‐Toprak:** Visualization (equal); writing – review and editing (equal). **Carmen Jochem:** Conceptualization (equal); writing – review and editing (equal). **Michael F. Leitzmann:** Supervision (lead); writing – review and editing (equal). **Hansjörg Baurecht:** Conceptualization (equal); data curation (equal); formal analysis (equal); methodology (equal); project administration (equal); validation (equal); visualization (equal); writing – original draft (equal).

## ETHICS STATEMENT

All primary studies included in this meta‐analysis received ethical approval.

## Supporting information


**Appendix S1:** Supporting Information

## Data Availability

Data are available upon request.
