# Supplementary material for: Anthropometric factors and the risk of ovarian cancer: A systematic review and meta‐analysis
Source: Cancer Rep (Hoboken). 2022 Apr 5;5(11):e1618. doi: 10.1002/cnr2.1618 (PMC9675384; doi:10.1002/cnr2.1618)
Supplement: Supplementary file 1 — Appendix S1: Supporting Information [file CNR2-5-e1618-s001.docx]

**Supporting Information**

**Article title:** Anthropometric factors and the risk of ovarian cancer: a systematic review and meta-analysis

**Journal:** Cancer Reports

**Authors:** Bernadette Ellwanger, Susanne Schüler-Toprak, MD; Carmen Jochem, MD; Michael F. Leitzmann, MD; Hansjörg Baurecht, PhD

1

**Supporting Figure 1 a** Forest plot BMI at young adulthood overweight **b** Forest plot BMI at young adulthood obesity


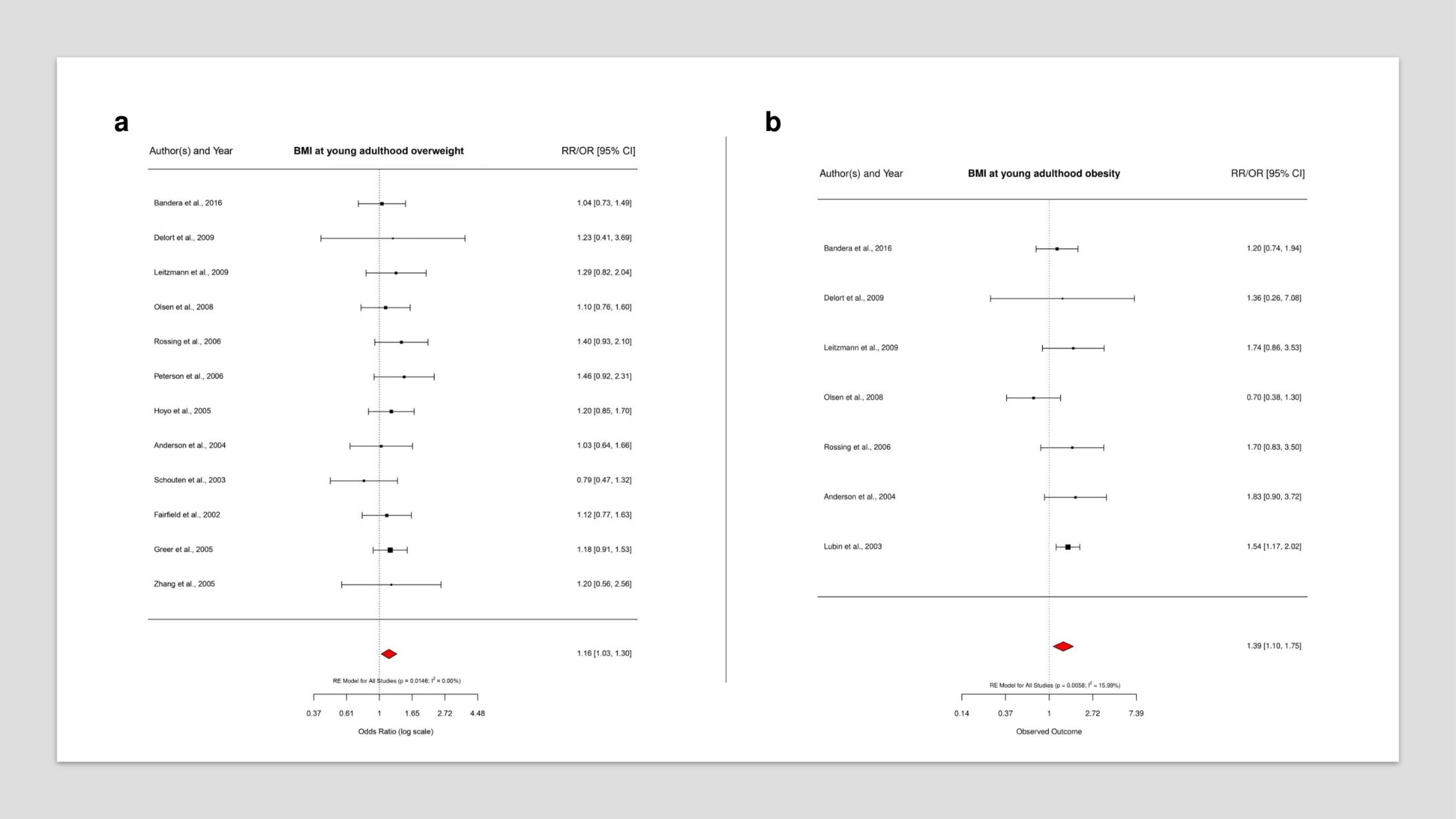


**Supporting Figure 2 a** Forest plot premenopausal overweight and ovarian cancer **b** Forest plot premenopausal obesity and ovarian cancer


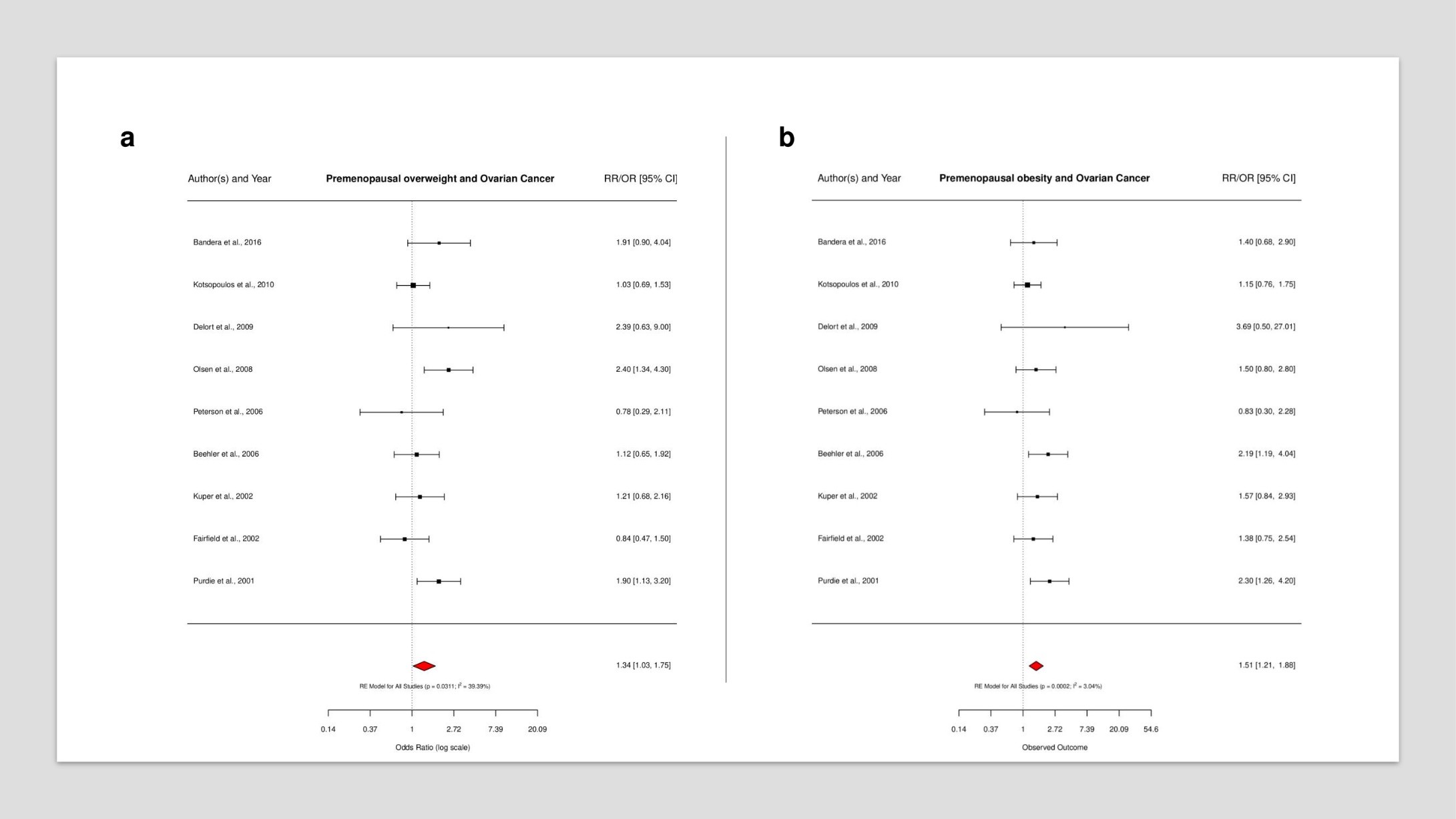


**Supporting Figure 3 a** Forest plot postmenopausal overweight and ovarian cancer **b** Forest plot postmenopausal obesity and ovarian cancer


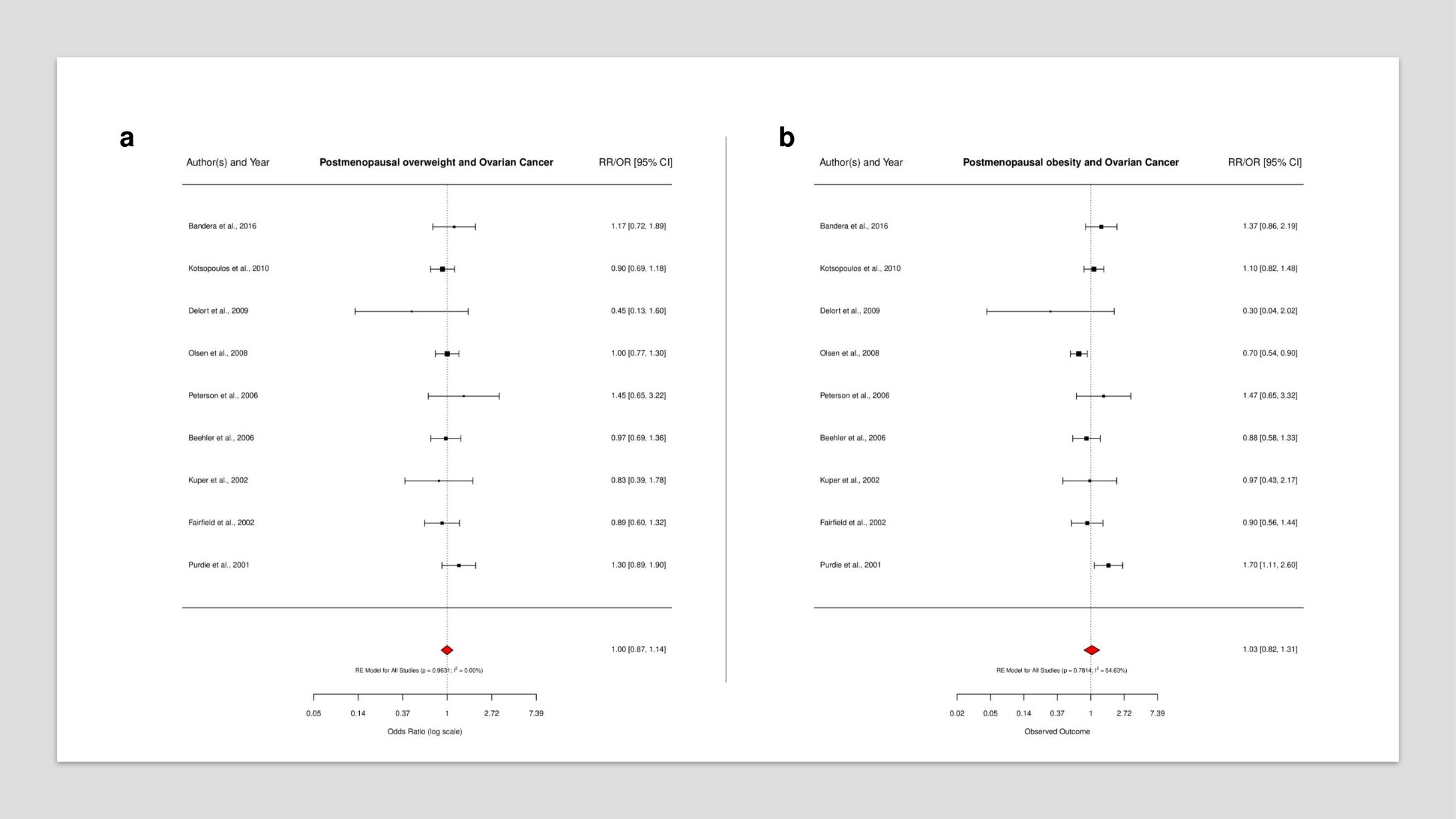


**Supporting Figure 4 a** Funnel plot overweight and ovarian cancer, **b** Trim and Fill overweight, **c** Funnel plot obesity, **d** Trim and Fill obesity


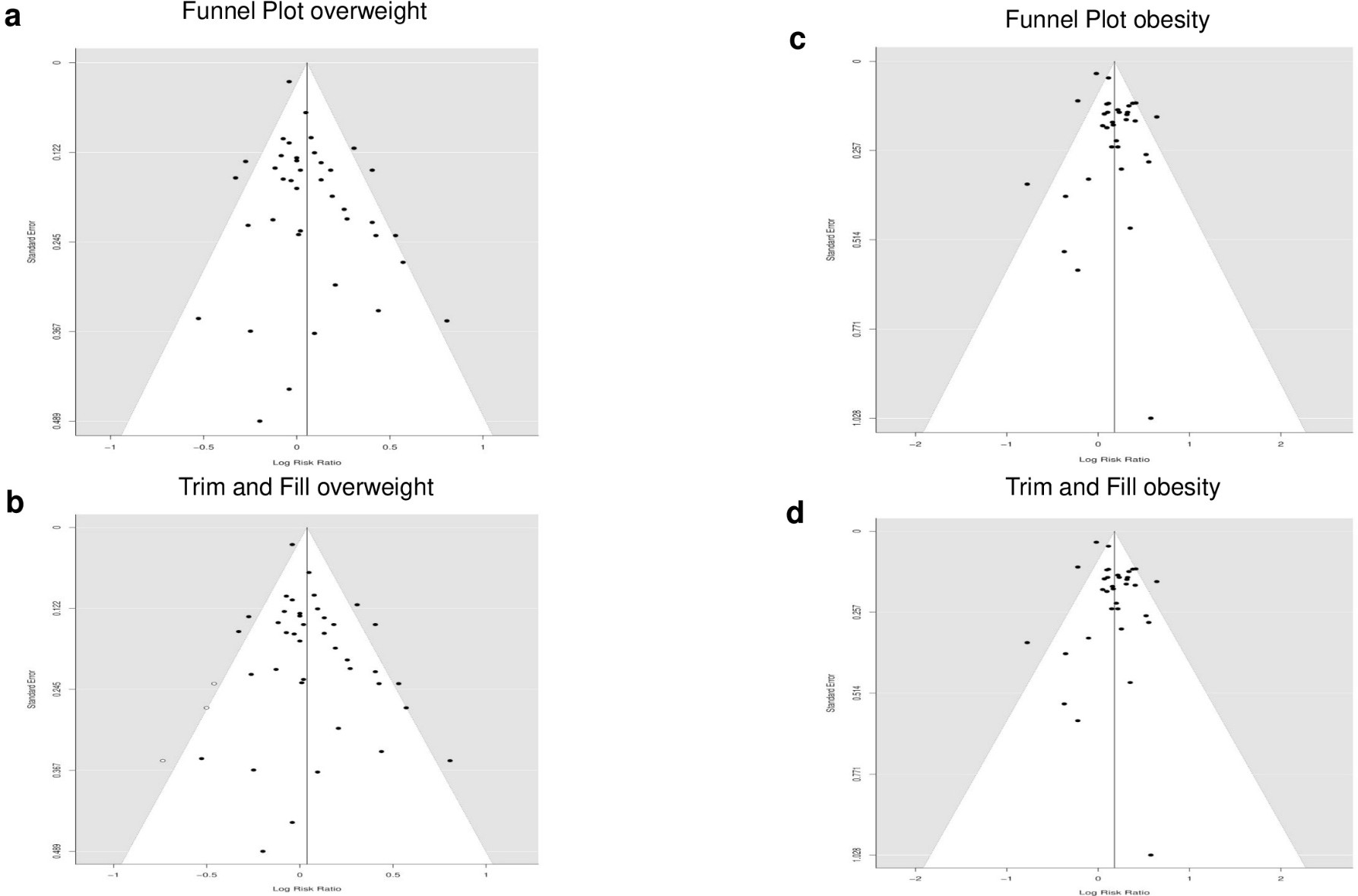


**Supporting Table S1** Study characteristics of the included 26 case-control and 26 cohort studies of overweight, obesity and ovarian cancer risk

| **Case-control studies** | | | | | | | | | |
| --- | --- | --- | --- | --- | --- | --- | --- | --- | --- |
| **Author, Year** | **PMID/DOI** | **Country** | **Study name** | **Ovarian**  **cancer subtype** | **Age** | **Cases/ Controls** | **Case ascertainment** | **Control ascertainment** | **Exposure measure** |
| **Nunez et al., 2017(1)** | 10.1016/j.cane2  017.01.002 | Australia | The Cancer Lifestyle and Evaluation of risk Study (CLEAR) | total | >18 | 151/967 | participants with self- reported incidence OC  cancer | Cancer free partners of cases in this study(sex-matched) | Self-reported |
| **Bandera et al., 2016(2)** | 27038123 | USA | The African American Cancer Epidemiology  Study (AACES) | epithelial | 20-79 | 492/696 | state cancer registries | Random digit dialing (RDD) | Self-reported |
| **Schildkraut et al., 2014(3)** | 25242549 | USA | The African American Cancer Epidemiology Study (AACES) | Epithelial | 20-79 | 403/639 | state cancer registries, SEER registries, gynecologic oncology  departments | RDD | Self-reported |
| **Su et al., 2012(4)** | 23065073 | China |  | Epithelial+ histological subtypes | >75 | 500/500 | Recruited from public hospitals (The Overseas Hospital), Zhujiang Hospital, General Hospital of Guangzhou Military Command, Second Affiliated Hospital  of Zhongshan University | recruited from same hospitals of the departments of ophthalmology, orthopedics,  respiratory disease, gastroenterology, and  physiotherapy. | Self-reported |
| **Moorman et al.,**  **2009(6)** | 19605513 | USA | The North Carolina  Ovarian Cancer Study | Epithelial | 20-74 | 143/189  (afro-american) | North Carolina State  Cancer registry | RDD | Measured |
| **Moorman et al.,**  **2009(6)** | 19605513 | USA | The North Carolina  Ovarian Cancer Study | Epithelial | 20-74 | 943/868 (white population) | North Carolina State  Cancer registry | RDD | Measured |
| **Olsen et al.,2008(7)** | 18449887 | Australia | The Australian Ovarian  Cancer Study | Epithelial +histologic  subtypes | 18-79 | 1269/1509 | recruited in treatment Clinics, physicians, State Cancer Registries | Electoral Roll | Self-reported |
| **Soegaard et al., 2007(8)** | 17548679 | Denmark | MALOVA Study | Epithelial +  Histologic subtypes | 35-79 | 554/1564 | 16 gynecologic departments in Denmark | Computerized civil registration system | Self-reported |
| **Beehler et al., 2006(9)** | 17056817 | USA | - | Epithelial | - | 427/854 | Roswell Park Center Institute Tumor registry | Women who were presented at Roswell park center institute, but were not diagnosed with cancer | Self-reported |
| **Rossing et al.,**  **2006(10)** | 16633919 | USA | - | Epithelial | 35-54 | 355/1637 | SEER registries in  Atlanta, Detroit, Seattle | RDD between women the  CARE study | Self- reported |
| **Peterson et al. 2006(11)** | 16596298 | USA | - | Epithelial | 40-79 | 700/5943 | State cancer registries in  Wisconsin and Massachusetts | Randomly selected from lists if licensed drivers >65 and medicare beneficiaries from the center of medicare | Self-reported |
| **Greer et al,2005(12)** | 16596653 | USA | SHARE Study | Epithelial | 20-69 | 762/1348 | 39 hospitals around  Delaware Valley | RDD | Self-reported |
| **Hoyo et al., 2005(13)** | 16132804 | USA | North Carolina Ovarian Cancer Study (NCOCS) | Epithelial | 20-74 | 593/628 | Case Ascertainment System from the North Caroline State Cancer  Registry | RDD | Self-reported |
| **Zhang et al., 2005(14)** | 15979697 | China | - | Epithelial | <75 | 254/652 | patients at Zhejiang Hospital and nine other  general hospital | Zhejiang hospital (visitors, outpatients), women from the community | Self-reported |
| **Riman et al., 2004(15)** | 15648594 | Sweden | - | Epithelial+  histologic subtypes | 50-74 | 655/3899 | Regional Tumor registries | National population registry | Self-reported |

| **Pike et al., 2004(16)** | 15237010 | USA | - | Total | 18-74 | 477/660 | Cancer surveillance program of Los Angeles  County | Neighborhood control or  random Sample of Los Angeles County | Self-reported |
| --- | --- | --- | --- | --- | --- | --- | --- | --- | --- |
| **Lubin et al., 2003(17)** | 12522018 | Israel | - | Epithelial | - | 1269/2111 | 22 gynecological centers in Israel | Central population registry | Self- reported |
| **Dal Maso et al., 2002(18)** | 12175694 | Italy | Italian Multicenter case-  control study | Epithelial | 17-79 | 1031/2411 | From the greater teaching hospitals in Pordenone, Padua, Gorizia, Milan | Same geographical area, admitted to the hospitals with diagnoses not related to ovarian cancer | Self-reported |
| **Kuper et al., 2002(19)** | 12146850 | USA | - | Epithelial+ histologic subtypes | - | 563/523 | From hospital tumor boards and state cancer  registries in Massachusetts and New Hampshire | RDD and selection from  community lists | Self-reported |
| **Lukanova et al.,2002(20)** | 11992553 | Sweden | Multicenter nested Case control study three cohorts (NYUWHS, NSHDS, ORDET) | Epithelial | 30-70 | 122/233 | all cohort members with primary invasive epithelial OC diagnosed > 12 month after recruitment | two controls for each subject out of the cohorts matching the case | Measured (ORDET study  and 77% NSHDS), self-  reported (NYUWHS and  remaining  NSHDS) |
| **Purdie et al., 2001(21)** | 10.1023/A:10122  67619561 | Australia | Australian case-control  study | Epithelial + histologic  subtypes | 18-79 | 775/846 | From Gynecological oncology centers | State electoral roll | Self-reported |
| **Hirose et al.,1999(22)** | 10359038 | Japan | - | Total | >30 | 99/25488 | first-visit patients to the Aichi Cancer Center Hospital, hospital-based  cancer registry files | First visit outpatients, who had not been diagnosed with any type of cancer | Self-reported |
| **Ness et al.,2000(23)** | 10933270 | USA | - | Epithelial | 20-69 | 767/1367 | From 39 hospitals in  Delaware Valley | RDD and health care  Financing Administration lists | Self-reported |
| **Mori et al., 1998(24)** | 9600117 | Japan | - | Epithelial | 32-84 | 89/323 | registered at 3 major gynecological oncological hospitals in Chikugo- Saga Counties of northern Kuyushu | participants of uterine cancer screening test in 2 cities and 1 town, same region | Self-reported |
| **Farrow et al., 1989(25)** | 2729264 | USA | - | Epithelial + histological subtypes | 35-74 | 277/655 | population-based registries in King or Pierce county, Washington, Salt Lake,Weber, Utah, Davis County, Utah | same County’s, household  surveys | Self-reported |
| **Cohort studies** | | | | | | | | | |
| **Author, Year** | **PMID/DOI** | **Country** | **Study name** | **Ovarian**  **cancer subtype** | **Age** | **Cases** | **Cohort Size** | **Year of Study** | **Exposure measure** |
| **Yang et al., 2012(26)** | 21960414 | USA | The National Institutes of Health (NIH)-AARP Diet and Health Study (NIH-  AARP) | Epithelial+ histologic subtypes | 50-71 | 849 | 169391 | 1995-2006 | Self-reported |
| **Bjᴓrge et al., 2011(27)** | 21984693 | Austria, Norway,  Sweden | metabolic Syndrome and Cancer Project (Me-Can Project) | Epithelial | - | 644 | 287320 | 1974-2005 | Measured |
| **Brändstedt et al.,2011(28)** | 21288792 | Sweden | Malmö Diet and Cancer  Study (MDCS) | Epithelial + histologic  subtypes | - | 93 | 17035 | 1991-2007 | Measured |
| **Kotstopolus et al.,2010(29)** | 20035276 | USA | Nurses’ Health Study I and II (NHS I, NHS II) | Epithelial | 30-55 (NHS  I) 25-42 (NHSII) | 862 | 121.700 (NHS I) 116.430 (NHS II) | 1976-2006 (NHSI) 1986-2005 (NHS II) | Self-reported |
| **Leitzmann et al.2009(30)** | 19127552 | USA | The National Institutes of Health (NIH)-AARP Diet and Health Study (NIH-  AARP) | Epithelial | 50-71 | 303 | 94525 | 1996-2003 | Self-reported |
| **Reeves et al., 2007(31)** | 17986716 | England,  Scotland | The Million Women Study | Total | 50-64 | 2406 | 1.2 Million | 1996-2001 | Self-reported |
| **Lundqvist et al., 2007(32)** | 17455257 | Sweden, Finland | - | Total | 43-83  (Sweden)  46-96  (Finland), | 151 old | 24 821 (older subjects) | 1969-2001 Sweden 1976-  2004 Finland | Self-reported |
| **Lundqvist et al., 2007(32)** | 17455257 | Sweden, Finland | - | Total | 18-47  (Sweden) 18-<46  (Finland) | 162 young | 43 328 (younger  subjects) | 1969-2001 Sweden 1976-  2004 Finland | Self-reported |
| **Lacey et al., 2006(33)** | 17027285 | USA | Breast Cancer Detection Demonstration Project Follow up Study (BCDDP) | Total | 31-89 | 346 | 46 026 | 1979-1998 | Measured |
| **Lukanova et. 2006(34)** | 10.1002/ijc.21354 | Sweden | The Northern Sweden  Health and Disease Cohort (NSHDC) | Epithelial | - | 90 | 35 362 | 1985-2003 | Measured |
| **Anderson et al.,**  **2004(35)** | 15042687 | USA | The Iowa’s Women’s  Health Study | Epithelial | 55-69 | 223 | 41 836 | 1986-2000 | Measured |
| **Kuriyama et al.,**  **2004(36)** | 15386435 | Japan | - | total | >40 | 5 | 15 054 | 1984-1992 | Self-reported |
| **Niwa et al., 2004(37)** | 16176517 | Japan | Japan Collaborative Cohort Study for the Evaluation of Cancer Risk (JACC) | Total | 40-79 | 38 | 36 456 | 1988-1999 | Self-reported |
| **Engeland et al.,**  **2003(38)** | 12928351 | Norway | - | Total | 14-74 | 7882 | 106 7354 | 1963-1999 | Measured |
| **Schouten et al.,**  **2003(39)** | 12615607 | Netherlands | The Netherlands Cohort  Study on Diet and Cancer | Epithelial | 55-69 | 172 | 62 573 | 1986-1993 | Self-reported |
| **Fairfield et al.,**  **2002(40)** | 12151152 | USA | Nurse`s Health Study | Epithelial | 30-55 | 402 | 109 445 | 1976-1996 | Self-reported |

**Supporting Table S2** Results random-effects meta-analyses and heterogeneity analyses including Stratification criteria, Number of included studies, RR, relative risk; RR, relative risk; CI, confidence interval; I2, heterogeneity among studies; P-heterogeneity, , P-value for test of random heterogeneity.

|  | **overweight** | | | | | **obesity** | | | | |
| --- | --- | --- | --- | --- | --- | --- | --- | --- | --- | --- |
| **Subgroups** | **N of studies** | **RR** | **95% CI** | **I²** | **Pheterogeneity** | **N of studies** | **RR** | **95% CI** | **I²** | **Pheterogeneity** |
| total incident ovarian cancer | 41(26 CC/15 cohort) | 1.06 | 1.00-1.12 | 37.78% | 0.0058 | 35(20CC/15 cohort) | 1.19 | 1.11-1.28 | 51.99% | 0.0001 |
| WHO BMI category | 34(20 CC/14 cohort) | 1.02 | 0.97-1-07 | 9.37% | 0.2695 | 30(16CC/14 cohort) | 1.17 | 1.08-1.25 | 42.85% | 0.0162 |
| **Life period of BMI measurement** | | | | | | | | | | |
| high BMI in young adulthood | 12 (10 CC/ 2 cohort) | 1.16 | 1.03-1.30 | 0.00% | 0.9348 | 7 (5 CC/ 2 cohort) | 1.39 | 1.10-1.75 | 15.99% | 0.3328 |
| high premenopausal BMI | 9 (7 CC/ 2 cohort) | 1.34 | 1.03-1.75 | 39.39% | 0.1128 | 9 (7 CC/ 2 cohort) | 1.51 | 1.21-1.88 | 3.04% | 0.5184 |
| high postmenopausal BMI | 9 (7 CC/ 2 cohort) | 1.00 | 0.87-1.14 | 0.00% | 0.0165 | 9 (7 CC/ 2 cohort) | 1.03 | 0.82-1.31 | 54.63% | 0.0165 |
| **Ovarian cancer subtype** | | | | | | | | | | |
| Ovarian cancer total | 12 (4 CC/ 8 cohort) | 1.04 | 0.95-1.14 | 28.11% | 0.0772 | 10 (4CC/ 6 cohort) | 1.09 | 0.97-1.23 | 40.85% | 0.1332 |
| Ovarian cancer epithelial | 29 (21 CC/ 8 cohort) | 1.06 | 0.98-1.14 | 36.65% | 0.0226 | 25 (16 CC/ 9 cohort | 1.22 | 1.12-1.33 | 44.51% | 0.0064 |
| **Histological subtypes of epithelial ovarian cancer** | | | | | | | | | | |
| mucinous subtype | 6 (6 CC) | 1.21 | 0.90-1.63 | 0.00% | 0.7634 | 5 (4 CC/ 1 cohort) | 1.44 | 1.03-2.01 | 0.00% | 0.3283 |
| endometroid subtype | 6 (5 CC/ 1 cohort) | 1.26 | 0.99-1.60 | 0.00% | 0.5595 | 6 (4 CC/ 2 cohort) | 1.24 | 0.96-1.60 | 0.00% | 0.6915 |
| serous subtype | 7 (6 CC/ 1 cohort) | 1.13 | 0.88-1.46 | 61.60% | 0.0243 | 6 (4 CC/ 2 cohort) | 1.12 | 0.84-1.50 | 68.71% | 0.0115 |
| clear cell subtype | 2 (2 CC) | 1.66 | 0.98-2.82 | 0.00% | 0.5432 | 4 (3 CC/ 1 cohort) | 1.82 | 1.11-2.99 | 24.25% | 0.2114 |
| undifferentiated subytpe | 3 (3 CC) | 1.05 | 0.52-2.11 | 56.65% | 0.0994 | 3 (2 CC/ 1 cohort) | 1.57 | 1.22-2.03 | 0.00% | 0.8323 |

| **Study type** | **overweight** | | | | | **obesity** | | | | |
| --- | --- | --- | --- | --- | --- | --- | --- | --- | --- | --- |
| **Subgroups** | **N of studies**  15 | **RR**  1.02 | **95% CI**  0.95-1.09 | **I²**  21.06% | **Pheterogeneity**  0.0567 | **N of studies**  15 | **RR**  1.10 | **95% CI**  1.02-1.19 | **I²**  26.41% | **Pheterogeneity**  0.2431 |
| Cohort studies |  |  |  |  |  |  |  |  |  |  |
| Case-Control studies | 26 | 1.08 | 0.99-1.17 | 33.97% | 0.0446 | 20 | 1.25 | 1.12-1.39 | 50.81% | 0.0022 |
| **Geographic region** | | | | | | | | | | |
| Europe | 13 (5 CC/ 8 Cohort) | 1.00 | 0.91-1.10 | 50.86% | 0.0254 | 12 (4 CC/ 8 cohort) | 1.08 | 0.97-1.20 | 39.27% | 0.0212 |
| Asia | 7 (5 CC/ 2 Cohort) | 1.32 | 1.04-1.66 | 29.61% | 0.2063 | 2 (1 CC/ 1 cohort) | 1.51 | 1.20-1.91 | 0.00% | 0.8737 |
| North America | 18 (12 CC/ 6 Cohort) | 1.04 | 0.97-1.12 | 0.00% | 0.7814 | 18 (12CC/ 6 cohort) | 1.23 | 1.15-1.32 | 0.00% | 0.9453 |
| Australia | 3 (3 CC) | 1.11 | 0.79-1.58 | 73.29% | 0.0358 | 3 (3 CC) | 1.22 | 0.73-2.04 | 87.30% | <0.0001 |
| **Adjustment factor(s)** | | | | | | | | | | |
| Adjusted for age | 38 (25 CC/ 13 cohort) | 1.05 | 0.99-1.11 | 32.94% | 0.0114 | 32 (18 CC/ 14 cohort) | 1.19 | 1.10-1.28 | 52.45% | 0.0003 |
| age at menarche | 11 (6 CC/ 5 cohort) | 1.05 | 0.94-1.16 | 0.01% | 0.279 | 8 (4 CC/ 4 cohort) | 1.21 | 1.07-1.37 | 10.05% | 0.5779 |
| parity | 26 (15 CC/ 11 cohort) | 1.08 | 1.01-1-16 | 21.28% | 0.0731 | 22 (11 CC/ 11 cohort) | 1.19 | 1.07-1.31 | 54.99% | 0.0037 |
| contraceptives | 24 (17 CC/ 7 cohort) | 1.04 | 0.97-1.12 | 24.74% | 0.1059 | 21 (14 CC/ 7 cohort) | 1.27 | 1.18-1.36 | 7.93% | 0.5489 |
| menopausal status | 9 (5 CC/ 4 cohort) | 1.01 | 0.89-1.13 | 0.00% | 0.3447 | 6 (3 CC/ 3 cohort) | 1.14 | 0.90-1.43 | 56.00% | 0.0411 |
| menopausal hormone therapy | 9 (2 CC/ 7 cohort) | 1.03 | 0.95-1.11 | 0.00% | 0.9268 | 10(2 CC/ 8 cohort) | 1.16 | 1.09-1.24 | 0.00% | 0.7830 |
| tubal ligation | 10 (8 CC/ 2 cohort) | 1.07 | 0.95-1.20 | 24.55% | 0.3345 | 10 (8 CC/ 2 cohort) | 1.29 | 1.13-1.46 | 30.77% | 0.2493 |
| smoking status | 12 (4 CC/ 8 cohort) | 1.18 | 1.03-1.35 | 42.35% | 0.0294 | 10 (2 CC/ 8 cohort) | 1.11 | 0.86-1.44 | 70.18% | 0.0094 |
| Number of live birth | 7 (5 CC/ 2 cohort) | 1.05 | 0.96-1.16 | 0.00% | 0.8323 | 7 (5CC/ 2 cohort) | 1.17 | 1.07-1.26 | 2.46% | 0.6390 |
| family history of breast and/or  ovarian cancer | 19 (12 CC/ 7 cohort) | 1.06 | 0.95-1.19 | 37.62% | 0.0539 | 15 (9 CC/ 6 cohort) | 1.29 | 1.17-1.42 | 15.71% | 0.4736 |

| **Exposure measure** | **overweight** | | | | | **obesity** | | | | |
| --- | --- | --- | --- | --- | --- | --- | --- | --- | --- | --- |
| **Subgroups** | **N of studies** | **RR** | **95% CI** | **I²** | **Pheterogeneity** | **N of studies** | **RR** | **95% CI** | **I²** | **Pheterogeneity** |
| BMI self-reported | 31 (21 CC/ 10 cohort) | 1.09 | 1.02-1.17 | 29.81% | 0.0423 | 25 (15 CC/ 10 cohort) | 1.24 | 1.14-1.35 | 46.64% | 0.0168 |
| BMI measured | t10 (4 CC/ 6 cohort) | 0.96 | 0.92-1.01 | 0.02% | 0.2814 | 10 (4 CC/ 6 cohort) | 1.03 | 0.93-1.13 | 8.96% | 0.2037 |

**Supporting Table S3** Adjustment Factors

| **Author, year** | **number of adjustment factors** | **Age (31)** | **oral contraceptives (20)** | **parity (20)** | **Family_history of breast and or ovarian cancer (15)** | **region (11)** | **hormone therapy (10)** | **smoking (10)** | **education (8)** | **age at menarche (8)** | **tubal ligation (8)** | **number of live birth (7)** | **race (6)** | **menopausal status (6)** | **physical activity(4)** | **alcohol intake (3)** |
| --- | --- | --- | --- | --- | --- | --- | --- | --- | --- | --- | --- | --- | --- | --- | --- | --- |
| Nunez et al. , 2017(1) | 5 | x | x |  |  | x |  |  |  |  |  |  |  |  | x |  |
| Bandera et al., 2016(2) | 9 | x | x | x | x | x |  |  | x | x | x |  |  | x |  |  |
| Schildkraut et al., 2014(3) | 3 | x | x | x |  |  |  |  |  |  |  |  |  |  |  |  |
| Yang et al., 2012(26) | 4 | x | x | x |  |  | x |  |  |  |  |  |  |  |  |  |
| Brändstedt et al., 2011(28) | 7 |  | x | x |  |  | x | x | x | x |  |  |  | x |  |  |
| Kotsopoulos et al., 2010(29) | 9 |  | x | x | x |  | x |  |  | x | x |  |  | x |  |  |
| Moorman et al., 2009 afro american population (6) | 6 | x | x |  | x |  |  |  |  | x | x | x |  |  |  |  |
| Moorman et al., 2009 white population (6) | 6 | x | x |  | x |  |  |  |  | x | x | x |  |  |  |  |
| Delort et al., 2009(5) | 1 | x |  |  |  |  |  |  |  |  |  |  |  |  |  |  |
| Leitzmann et al., 2009(30) | 7 | x | x |  | x |  | x |  |  |  |  |  | x |  | x |  |
| Olsen et al., 2008 (7) | 4 | x |  | x |  |  |  |  | x |  |  |  |  |  |  |  |
| Reeves et al., 2007(31) | 9 | x |  | x |  | x | x | x |  |  |  | x |  |  |  | x |
| Lundqvist et al., 2007 old (32) | 8 | x |  | x |  | x |  | x |  |  |  |  |  |  | x |  |
| Lundqvist et al., 2007, young (32) | 9 | x |  | x |  | x |  | x |  |  |  |  |  |  | x |  |
| Soegaard et al., 2007(8) | 4 | x | x |  |  |  |  |  |  |  |  | x |  |  |  |  |
| Rossing et al., 2006(10) | 5 | x | x |  |  | x |  |  |  |  |  | x | x |  |  |  |
| Peterson et al., 2006(11) | 7 | x | x | x | x | x |  |  | x |  | x |  |  |  |  |  |
| Lacey et al., 2006(33) | 7 | x | x | x |  |  | x |  |  |  |  |  | x | x |  |  |
| Beehler et al., 2006(9) | 3 | x |  |  |  | x |  |  |  |  |  |  |  |  |  |  |
| Hoyo et al., 2005(13) | 7 | x | x | x | x |  |  |  |  |  |  |  | x |  |  |  |
| Zhang et al., 2005(14) | 11 | x | x | x | x |  |  | x |  |  |  |  |  | x |  |  |
| Lukanova et al., 2006(34) | 3 | x |  |  | x |  |  | x |  |  |  |  |  |  |  |  |
| Riman et al., 2004(15) | 5 | x | x | x |  |  | x |  |  | x |  |  |  |  |  |  |
| Pike et al., 2004(16) | 19 | x | x | x | x |  | x |  | x |  | x | x | x | x |  |  |
| Niwa et al. 2004(37) | 9 | x |  | x | x | x |  | x | x | x |  |  |  |  |  | x |
| Anderson et al., 2004(35) | 7 | x |  |  | x |  | x | x |  |  |  | x |  |  |  |  |
| Kuriyama et al., 2004(36) | 13 | x |  |  |  |  |  | x |  | x |  |  |  |  |  | x |
| Engeland et al., 2003(38) | 2 | x |  |  |  |  |  |  |  |  |  |  |  |  |  |  |
| Schouten et al., 2003(39) | 5 | x | x | x |  |  | x |  |  |  |  |  |  |  |  |  |
| Dal Maso et al., 2002(18) | 5 | x | x | x |  | x |  |  | x |  |  |  |  |  |  |  |
| Kuper et al., 2002(19) | 8 | x | x | x | x | x |  |  | x |  | x |  |  |  |  |  |
| Fairfield et al., 2002 | 6 | x | x | x |  |  |  | x |  | x | x |  |  |  |  |  |
| Ness et al., 2000 | 4 | x |  | x | x |  |  |  |  |  |  |  | x |  |  |  |
| Hirose et al., 1999 | 5 | x |  | x | x |  |  |  |  |  |  |  |  |  |  |  |

| **Author, year** | **height (2)** | **socioeconomic status (2)** | **diabetes (2)** | **pregnancy (2)** | **marital status (2)** | **caffeine intake (1)** | **hysterectomy (1)** | **number of additional pregnancies (1)** | **enrollment period (1)** | **calendar time (1)** | **year of study participation (1)** | **total energy intake (1)** | **nulliparity (1)** | **use of genital talk (1)** | **age at last birth (1)** | **oophorectomy (1)** | **birth cohort (1)** | **age at full birth pregnancy (1)** | **menstrual irregularity (1)** |
| --- | --- | --- | --- | --- | --- | --- | --- | --- | --- | --- | --- | --- | --- | --- | --- | --- | --- | --- | --- |
| Nunez et al. , 2017 |  |  |  |  |  |  | x |  |  |  |  |  |  |  |  |  |  |  |  |
| Bandera et al., 2016 |  |  |  |  |  |  |  |  |  |  |  |  |  |  |  |  |  |  |  |
| Schildkraut et al., 2014 |  |  |  |  |  |  |  |  |  |  |  |  |  |  |  |  |  |  |  |
| Yang et al., 2012 |  |  |  |  |  |  |  |  |  |  |  |  |  |  |  |  |  |  |  |
| Brandstedt et al., 2011 |  |  |  |  |  |  |  |  |  |  |  |  |  |  |  |  |  |  |  |
| Kotsopoulos et al., 2010 | x |  |  |  |  | x |  |  |  |  |  |  |  |  |  |  |  |  |  |
| Moorman et al., 2009 |  |  |  |  |  |  |  |  |  |  |  |  |  |  |  |  |  |  |  |
| Moorman et al., 2009 |  |  |  |  |  |  |  |  |  |  |  |  |  |  |  |  |  |  |  |
| Delort et al., 2009 |  |  |  |  |  |  |  |  |  |  |  |  |  |  |  |  |  |  |  |
| Leitzmann et al., 2009 |  |  |  |  |  |  |  |  |  |  |  |  |  |  |  |  |  |  |  |
| Olsen et al., 2008 |  |  |  |  |  |  |  |  |  |  |  |  |  |  |  |  |  |  |  |
| Reeves et al., 2007 |  | x |  |  |  |  |  |  |  |  |  |  |  |  |  |  |  |  |  |
| Lundqvist et al., 2007 |  |  | x |  |  |  |  |  |  |  |  |  |  |  |  |  |  |  |  |
| Lundqvist et al., 2007 |  |  | x |  |  |  |  |  |  |  |  |  |  |  |  |  |  |  |  |
| Soegaard et al., 2007 |  |  |  | x |  |  |  |  |  |  |  |  |  |  |  |  |  |  |  |
| Rossing et al., 2006 |  |  |  |  |  |  |  | x |  |  |  |  |  |  |  |  |  |  |  |
| Peterson et al., 2006 |  |  |  |  |  |  |  |  | x |  |  |  |  |  |  |  |  |  |  |
| Lacey et al., 2006 |  |  |  |  |  |  |  |  |  | x |  |  |  |  |  |  |  |  |  |
| Beehler et al., 2006 |  |  |  |  |  |  |  |  |  |  | x |  |  |  |  |  |  |  |  |
| Hoyo et al., 2005 |  |  |  |  |  |  |  |  |  |  |  |  |  |  |  |  |  |  |  |
| Zhang et al., 2005 |  |  |  |  |  |  |  |  |  |  |  | x |  |  |  |  |  |  |  |
| Lukanova et al., 2005 |  |  |  |  |  |  |  |  |  |  |  |  |  |  |  |  |  |  |  |
| Riman et al., 2004 |  |  |  |  |  |  |  |  |  |  |  |  |  |  |  |  |  |  |  |
| Pike et al., 2004 |  | x |  | x |  |  |  |  |  |  |  |  | x | x | x |  |  |  |  |
| Niwa et al. 2004 |  |  |  |  |  |  |  |  |  |  |  |  |  |  |  |  |  |  |  |
| Anderson et al., 2004 |  |  |  |  |  |  |  |  |  |  |  |  |  |  |  | x |  |  |  |
| Kuriyama et al., 2004 |  |  |  |  |  |  |  |  |  |  |  |  |  |  |  |  |  |  |  |
| Engeland et al., 2003 |  |  |  |  |  |  |  |  |  |  |  |  |  |  |  |  | x |  |  |
| Schouten et al., 2003 | x |  |  |  |  |  |  |  |  |  |  |  |  |  |  |  |  |  |  |
| Dal Maso et al., 2002 |  |  |  |  |  |  |  |  |  |  |  |  |  |  |  |  |  |  |  |
| Kuper et al., 2002 |  |  |  |  | x |  |  |  |  |  |  |  |  |  |  |  |  |  |  |
| Fairfield et al., 2002 |  |  |  |  |  |  |  |  |  |  |  |  |  |  |  |  |  |  |  |
| Ness et al., 2000 |  |  |  |  |  |  |  | x |  |  |  |  |  |  |  |  |  |  |  |
| Hirose et al., 1999 |  |  |  |  | x |  |  |  |  |  |  |  |  |  |  |  |  | x | x |

**Supporting Table S4** Results influence diagnostic overweight

| **BMI**  **overweight** |  |  |  |  |  |  |  |  |
| --- | --- | --- | --- | --- | --- | --- | --- | --- |
|  | RR | Lower CI | Upper CI | P-value | Q | PQ-statistic | tau^2^ | I2 |
| 1 | 1.06 | 1.00 | 1.13 | 0.0481 | 64.6 | 0.0061 | 0.0103 | 38.1 |
| 2 | 1.05 | 0.99 | 1.12 | 0.0906 | 64.6 | 0.0061 | 0.0102 | 37.8 |
| 3 | 1.05 | 0.99 | 1.12 | 0.0897 | 64.6 | 0.0061 | 0.0101 | 37.8 |
| 4 | 1.05 | 0.99 | 1.11 | 0.1013 | 61.9 | 0.0113 | 0.0091 | 35.3 |
| 5 | 1.06 | 1.00 | 1.12 | 0.0665 | 66.1 | 0.0043 | 0.0109 | 39.5 |
| 6 | 1.06 | 1.01 | 1.13 | 0.0317 | 61.4 | 0.0125 | 0.0081 | 32.4 |
| 7 | 1.06 | 1.00 | 1.13 | 0.0552 | 65.9 | 0.0045 | 0.0115 | 40.0 |
| 8 | 1.06 | 1.00 | 1.12 | 0.066 | 66.1 | 0.0043 | 0.0105 | 38.8 |
| 9 | 1.06 | 1.00 | 1.13 | 0.0506 | 65.6 | 0.0049 | 0.011 | 39.2 |
| 10 | 1.06 | 1.00 | 1.12 | 0.0593 | 65.6 | 0.0048 | 0.0105 | 38.0 |
| 11 | 1.06 | 1.00 | 1.13 | 0.0489 | 65.3 | 0.0052 | 0.0107 | 38.7 |
| 12 | 1.06 | 0.99 | 1.12 | 0.081 | 65.6 | 0.0048 | 0.0115 | 40.3 |
| 13 | 1.06 | 0.99 | 1.13 | 0.075 | 65.8 | 0.0047 | 0.0127 | 40.6 |
| 14 | 1.05 | 0.99 | 1.12 | 0.093 | 64.7 | 0.0060 | 0.0106 | 38.4 |
| 15 | 1.05 | 0.99 | 1.11 | 0.1025 | 62.8 | 0.0092 | 0.0092 | 35.6 |
| 16 | 1.06 | 1.00 | 1.13 | 0.0487 | 65.5 | 0.0050 | 0.011 | 38.8 |
| 17 | 1.05 | 0.99 | 1.12 | 0.093 | 64.7 | 0.0060 | 0.0106 | 38.4 |
| 18 | 1.06 | 0.99 | 1.12 | 0.0759 | 65.7 | 0.0048 | 0.0105 | 38.7 |

| 19 | 1.06 | 1.00 | 1.13 | 0.0636 | 66.1 | 0.0043 | 0.0116 | 40.5 |
| --- | --- | --- | --- | --- | --- | --- | --- | --- |
| 20 | 1.06 | 1.00 | 1.13 | 0.0669 | 66.1 | 0.0043 | 0.0115 | 40.4 |
| 21 | 1.06 | 1.00 | 1.13 | 0.0638 | 66.1 | 0.0043 | 0.0116 | 40.5 |
| 22 | 1.06 | 1.00 | 1.13 | 0.0559 | 65.7 | 0.0047 | 0.0108 | 39.0 |
| 23 | 1.06 | 0.99 | 1.12 | 0.0858 | 65.3 | 0.0052 | 0.0111 | 39.6 |
| 24 | 1.05 | 0.99 | 1.11 | 0.0992 | 63.0 | 0.0088 | 0.0094 | 36.0 |
| 25 | 1.05 | 0.99 | 1.13 | 0.0796 | 65.7 | 0.0048 | 0.0119 | 40.7 |
| 26 | 1.06 | 1.00 | 1.13 | 0.0609 | 66.1 | 0.0044 | 0.0113 | 40.0 |
| 27 | 1.05 | 0.99 | 1.11 | 0.0972 | 61.0 | 0.0137 | 0.0092 | 35.6 |
| 28 | 1.05 | 0.99 | 1.12 | 0.082 | 65.5 | 0.0049 | 0.011 | 39.5 |
| 29 | 1.06 | 1.00 | 1.12 | 0.0635 | 65.9 | 0.0045 | 0.0105 | 38.7 |
| 30 | 1.07 | 1.00 | 1.14 | 0.0438 | 58.7 | 0.0221 | 0.011 | 30.0 |
| 31 | 1.05 | 0.99 | 1.12 | 0.0865 | 65.1 | 0.0054 | 0.0106 | 38.6 |
| 32 | 1.07 | 1.01 | 1.13 | 0.0301 | 61.6 | 0.012 | 0.0078 | 31.4 |
| 33 | 1.06 | 1.00 | 1.12 | 0.0671 | 66.1 | 0.0043 | 0.0109 | 39.5 |
| 34 | 1.06 | 1.00 | 1.13 | 0.0559 | 65.8 | 0.0046 | 0.0111 | 39.7 |
| 35 | 1.06 | 1.00 | 1.13 | 0.0648 | 66.1 | 0.0043 | 0.0112 | 40.0 |
| 36 | 1.04 | 0.99 | 1.10 | 0.1539 | 59.5 | 0.0189 | 0.0065 | 27.4 |
| 37 | 1.06 | 1.00 | 1.13 | 0.0504 | 63.7 | 0.0075 | 0.0102 | 38.1 |
| 38 | 1.04 | 0.99 | 1.10 | 0.1502 | 58.7 | 0.022 | 0.0063 | 27.2 |
| 39 | 1.06 | 1.00 | 1.12 | 0.0696 | 66.1 | 0.0044 | 0.0105 | 38.8 |
| 40 | 1.05 | 0.99 | 1.12 | 0.0836 | 64.5 | 0.0062 | 0.01 | 37.7 |
| 41 | 1.05 | 0.99 | 1.11 | 0.1092 | 61.2 | 0.013 | 0.0086 | 34.1 |

**Table S5** Results influence diagnostic obesity

| **BMI**  **obesity** |  |  |  |  |  |  |  |  |
| --- | --- | --- | --- | --- | --- | --- | --- | --- |
| study | RR | Lower CI | Upper CI | P-value | Q | PQ-statistic | tau^2^ | I2 |
| 1 | 1.20 | 1.11 | 1.29 | 0.0000 | 72.3 | 0.0001 | 0.0206 | 54.6 |
| 2 | 1.18 | 1.10 | 1.28 | 0.0000 | 67.3 | 0.0003 | 0.0192 | 52.1 |
| 3 | 1.19 | 1.10 | 1.29 | 0.0000 | 70.3 | 0.0001 | 0.0204 | 53.9 |
| 4 | 1.20 | 1.11 | 1.30 | 0.0000 | 72.1 | 0.0001 | 0.0201 | 54.1 |
| 5 | 1.20 | 1.11 | 1.30 | 0.0000 | 72.5 | 0.0001 | 0.0211 | 54.8 |
| 6 | 1.20 | 1.11 | 1.30 | 0.0000 | 72.5 | 0.0001 | 0.0213 | 54.7 |
| 7 | 1.19 | 1.11 | 1.29 | 0.0000 | 72.2 | 0.0001 | 0.02 | 54.1 |
| 8 | 1.20 | 1.11 | 1.30 | 0.0000 | 72.5 | 0.0001 | 0.0212 | 54.6 |
| 9 | 1.20 | 1.11 | 1.30 | 0.0000 | 71.7 | 0.0001 | 0.0199 | 54.0 |
| 10 | 1.19 | 1.10 | 1.29 | 0.0000 | 71.8 | 0.0001 | 0.0211 | 54.8 |
| 11 | 1.22 | 1.13 | 1.31 | 0.0000 | 63.8 | 0.0007 | 0.0135 | 43.2 |
| 12 | 1.20 | 1.10 | 1.31 | 0.0000 | 72.5 | 0.0001 | 0.0219 | 50.0 |
| 13 | 1.20 | 1.11 | 1.30 | 0.0000 | 71.1 | 0.0001 | 0.0197 | 53.7 |
| 14 | 1.20 | 1.11 | 1.29 | 0.0000 | 72.2 | 0.0001 | 0.0199 | 54.1 |
| 15 | 1.19 | 1.10 | 1.28 | 0.0000 | 69.4 | 0.0001 | 0.0196 | 53.1 |
| 16 | 1.20 | 1.11 | 1.29 | 0.0000 | 72.3 | 0.0001 | 0.0203 | 54.3 |
| 17 | 1.20 | 1.11 | 1.29 | 0.0000 | 72.5 | 0.0001 | 0.0206 | 54.6 |
| 18 | 1.20 | 1.11 | 1.30 | 0.0000 | 72.4 | 0.0001 | 0.021 | 54.9 |

| 19 | 1.19 | 1.10 | 1.29 | 0.0000 | 69.2 | 0.0001 | 0.0201 | 53.4 |
| --- | --- | --- | --- | --- | --- | --- | --- | --- |
| 20 | 1.19 | 1.10 | 1.29 | 0.0000 | 71.9 | 0.0001 | 0.0212 | 54.9 |
| 21 | 1.19 | 1.10 | 1.28 | 0.0000 | 70.1 | 0.0001 | 0.0195 | 53.4 |
| 22 | 1.19 | 1.10 | 1.29 | 0.0000 | 70.6 | 0.0001 | 0.0205 | 54.1 |
| 23 | 1.19 | 1.10 | 1.29 | 0.0000 | 71.0 | 0.0001 | 0.0205 | 54.3 |
| 24 | 1.20 | 1.11 | 1.29 | 0.0000 | 72.3 | 0.0001 | 0.0199 | 54.0 |
| 25 | 1.20 | 1.11 | 1.30 | 0.0000 | 72.4 | 0.0001 | 0.0209 | 54.9 |
| 26 | 1.22 | 1.13 | 1.31 | 0.0000 | 52.3 | 0.0132 | 0.0165 | 40.3 |
| 27 | 1.19 | 1.10 | 1.28 | 0.0000 | 70.1 | 0.0001 | 0.0195 | 53.3 |
| 28 | 1.20 | 1.11 | 1.30 | 0.0000 | 72.4 | 0.0001 | 0.0209 | 54.5 |
| 29 | 1.20 | 1.11 | 1.29 | 0.0000 | 72.3 | 0.0001 | 0.0205 | 54.5 |
| 30 | 1.20 | 1.11 | 1.30 | 0.0000 | 72.4 | 0.0001 | 0.0206 | 54.5 |
| 31 | 1.20 | 1.11 | 1.30 | 0.0000 | 72.5 | 0.0001 | 0.0208 | 54.7 |
| 32 | 1.18 | 1.09 | 1.28 | 0.0000 | 65.8 | 0.0004 | 0.0184 | 51.0 |
| 33 | 1.21 | 1.12 | 1.30 | 0.0000 | 66.2 | 0.0004 | 0.0183 | 51. |
| 34 | 1.17 | 1.09 | 1.26 | 0.0000 | 61.1 | 0.0014 | 0.0149 | 46.2 |

**Supporting Table S6** Results influence diagnostic for time points of measured BMI

| **BMI overweight at**  **young adulthood** |  |  |  |  |  |  |  |  |
| --- | --- | --- | --- | --- | --- | --- | --- | --- |
| study | RR | Lower CI | Upper CI | P-value | Q | PQ-statistic | tau^2^ | I2 |
| 1 | 1.17 | 1.04 | 1.33 | 0.0120 | 4.5 | 0.9201 | 0.0000 | 0.0 |
| 2 | 1.16 | 1.03 | 1.30 | 0.0157 | 4.9 | 0.8969 | 0.000 | 0.0 |
| 3 | 1.15 | 1.02 | 1.30 | 0.0253 | 4.7 | 0.9104 | 0.000 | 0.0 |
| 4 | 1.17 | 1.03 | 1.32 | 0.0161 | 4.8 | 0.9015 | 0.000 | 0.0 |
| 5 | 1.14 | 1.01 | 1.29 | 0.0398 | 4.0 | 0.9468 | 0.000 | 0.0 |
| 6 | 1.14 | 1.01 | 1.29 | 0.0361 | 3.9 | 0.9526 | 0.000 | 0.0 |
| 7 | 1.15 | 1.02 | 1.31 | 0.0261 | 4.9 | 0.899 | 0.000 | 0.0 |
| 8 | 1.16 | 1.03 | 1.31 | 0.0165 | 4.9 | 0.8967 | 0.000 | 0.0 |
| 9 | 1.15 | 1.01 | 1.32 | 0.0354 | 4.9 | 0.8977 | 0.000 | 0.0 |
| 10 | 1.17 | 1.03 | 1.32 | 0.0128 | 4.7 | 0.9119 | 0.000 | 0.0 |
| 11 | 1.18 | 1.05 | 1.34 | 0.0065 | 2.7 | 0.9883 | 0.000 | 0.0 |
| 12 | 1.16 | 1.03 | 1.32 | 0.0175 | 4.9 | 0.8985 | 0.000 | 0.0 |
| **BMI obesity at young**  **adulthood** |  |  |  |  |  |  |  |  |
| study | estimate | ci.lb | ci.ub | pval | Q | Qp | tau2 | I2 |
| 1 | 1.36 | 0.87 | 2.12 | 0.1789 | 5.9 | 0.2063 | 0.0984 | 39.1 |
| 2 | 1.30 | 0.92 | 1.85 | 0.1352 | 6.0 | 0.1982 | 0.0530 | 33.6 |
| 3 | 1.24 | 0.85 | 1.80 | 0.2741 | 5.2 | 0.2693 | 0.0567 | 30.1 |
| 4 | 1.49 | 1.10 | 2.03 | 0.0109 | 1.4 | 0.8395 | 0.0000 | 0.0 |
| 5 | 1.24 | 0.85 | 1.83 | 0.2633 | 5.3 | 0.2544 | 0.0612 | 32.8 |

| 6 | 1.22 | 0.85 | 1.76 | 0.2857 | 4.9 | 0.2991 | 0.0470 | 27.1 |
| --- | --- | --- | --- | --- | --- | --- | --- | --- |
| **BMI premenopausal**  **overweight** |  |  |  |  |  |  |  |  |
| study | estimate | ci.lb | ci.ub | pval | Q | Qp | tau2 | I2 |
| 1 | 1.29 | 0.98 | 1.72 | 0.0734 | 11.9 | 0.1029 | 0.0661 | 42.1 |
| 2 | 1.42 | 1.05 | 1.91 | 0.0221 | 11.1 | 0.135 | 0.0683 | 38.2 |
| 3 | 1.31 | 1.00 | 1.72 | 0.0509 | 12.2 | 0.0952 | 0.0633 | 42.7 |
| 4 | 1.22 | 0.97 | 1.54 | 0.0941 | 8.3 | 0.3067 | 0.0179 | 15.7 |
| 5 | 1.39 | 1.05 | 1.83 | 0.021 | 11.9 | 0.1043 | 0.0651 | 42.7 |
| 6 | 1.38 | 1.02 | 1.87 | 0.0377 | 12.6 | 0.0826 | 0.0820 | 45.4 |
| 7 | 1.36 | 1.00 | 1.85 | 0.0465 | 12.9 | 0.0748 | 0.0848 | 46.8 |
| 8 | 1.43 | 1.09 | 1.87 | 0.0108 | 10.4 | 0.1660 | 0.0511 | 34.7 |
| 9 | 1.26 | 0.96 | 1.67 | 0.1001 | 10.7 | 0.1525 | 0.0542 | 35.3 |
| **BMI premenopausal**  **obesity** |  |  |  |  |  |  |  |  |
| study | estimate | ci.lb | ci.ub | pval | Q | Qp | tau2 | I2 |
| 1 | 1.53 | 1.20 | 1.94 | 0.0005 | 7.1 | 0.4157 | 0.0117 | 9.7 |
| 2 | 1.65 | 1.29 | 2.12 | 0.0001 | 5.0 | 0.6560 | 0.0000 | 0.0 |
| 3 | 1.49 | 1.20 | 1.86 | 0.0003 | 6.4 | 0.4959 | 0.0029 | 2.9 |
| 4 | 1.52 | 1.19 | 1.94 | 0.0008 | 7.2 | 0.4115 | 0.0142 | 11.2 |
| 5 | 1.55 | 1.24 | 1.95 | 0.0001 | 5.8 | 0.5667 | 0.0054 | 5.0 |
| 6 | 1.43 | 1.14 | 1.80 | 0.002 | 5.5 | 0.5947 | 0.0000 | 0.0 |
| 7 | 1.51 | 1.18 | 1.93 | 0.001 | 7.2 | 0.4134 | 0.0136 | 10.7 |
| 8 | 1.54 | 1.20 | 1.97 | 0.0006 | 7.1 | 0.4206 | 0.0145 | 11.3 |
| 9 | 1.42 | 1.13 | 1.78 | 0.0027 | 5.0 | 0.6600 | 0.0000 | 0.0 |

| **BMI postmenopausal**  **overweight** |  |  |  |  |  |  |  |  |
| --- | --- | --- | --- | --- | --- | --- | --- | --- |
| study | estimate | ci.lb | ci.ub | pval | Q | Qp | tau2 | I2 |
| 1 | 0.98 | 0.86 | 1.13 | 0.8182 | 5.3 | 0.6215 | 0.0000 | 0.0 |
| 2 | 1.03 | 0.89 | 1.20 | 0.7136 | 5.1 | 0.6521 | 0.0000 | 0.0 |
| 3 | 1.01 | 0.88 | 1.15 | 0.9353 | 4.3 | 0.7504 | 0.0000 | 0.0 |
| 4 | 0.10 | 0.86 | 1.16 | 0.9574 | 5.8 | 0.5659 | 0.0000 | 0.0 |
| 5 | 0.99 | 0.86 | 1.13 | 0.8426 | 4.9 | 0.6712 | 0.0000 | 0.0 |
| 6 | 1.00 | 0.87 | 1.15 | 0.981 | 5.7 | 0.5694 | 0.0000 | 0.0 |
| 7 | 1.00 | 0.88 | 1.14 | 0.9711 | 5.6 | 0.5931 | 0.0000 | 0.0 |
| 8 | 1.01 | 0.88 | 1.16 | 0.8772 | 5.4 | 0.6087 | 0.0000 | 0.0 |
| 9 | 0.96 | 0.84 | 1.11 | 0.5849 | 3.6 | 0.8195 | 0.0000 | 0.0 |
| **BMI postmenopausal**  **obesity** |  |  |  |  |  |  |  |  |
| study | estimate | ci.lb | ci.ub | pval | Q | Qp | tau2 | I2 |
| 1 | 0.10 | 0.78 | 1.28 | 0.9677 | 16.5 | 0.0209 | 0.0626 | 55.5 |
| 2 | 1.02 | 0.78 | 1.35 | 0.8644 | 17.9 | 0.0124 | 0.0789 | 56.9 |
| 3 | 1.05 | 0.84 | 1.33 | 0.6721 | 17.2 | 0.016 | 0.061 | 57.9 |
| 4 | 1.13 | 0.93 | 1.38 | 0.2145 | 8.9 | 0.2576 | 0.0161 | 20.0 |
| 5 | 1.01 | 0.79 | 1.29 | 0.9332 | 17.7 | 0.0134 | 0.0634 | 57.9 |
| 6 | 1.06 | 0.81 | 1.39 | 0.655 | 18.4 | 0.0102 | 0.073 | 58.4 |
| 7 | 1.04 | 0.81 | 1.34 | 0.7607 | 18.7 | 0.0092 | 0.0683 | 59.7 |
| 8 | 1.06 | 0.81 | 1.37 | 0.6851 | 18.6 | 0.0096 | 0.0732 | 59.3 |
| 9 | 0.95 | 0.77 | 1.17 | 0.6301 | 11.4 | 0.1237 | 0.0321 | 38.4 |

**Supporting Table S7** Results influence diagnostic for different histologic subtypes

| **BMI overweight mucinous** | |  |  |  |  |  |  |  |  |  |
| --- | --- | --- | --- | --- | --- | --- | --- | --- | --- | --- |
| study | estimate | zval | pval | ci.lb | ci.ub | Q | Qp | tau2 | I2 | H2 |
| 1 | 1.24 | 1.3297 | 0.1836 | 0.90 | 1.69 | 2.4 | 0.6598 | 0.0000 | 0.0 | 1.0000 |
| 2 | 1.26 | 1.4419 | 0.1493 | 0.92 | 1.74 | 2.0 | 0.7274 | 0.0000 | 0.0 | 1.0000 |
| 3 | 1.00 | -0.011 | 0.9912 | 0.66 | 1.51 | 0.8 | 0.9389 | 0.0000 | 0.0 | 1.0000 |
| 4 | 1.23 | 1.2778 | 0.2013 | 0.90 | 1.68 | 2.5 | 0.6398 | 0.0000 | 0.0 | 1.0000 |
| 5 | 1.19 | 1.0149 | 0.3101 | 0.85 | 1.66 | 2.6 | 0.6309 | 0.0071 | 4.1 | 1.0427 |
| 6 | 1.27 | 1.4926 | 0.1355 | 0.93 | 1.73 | 1.6 | 0.8176 | 0.0000 | 0.0 | 1.0000 |
| **BMI obesity mucinous** | |  |  |  |  |  |  |  |  |  |
| study | estimate | zval | pval | ci.lb | ci.ub | Q | Qp | tau2 | I2 | H2 |
| 1 | 1.80 | 1.5912 | 0.1116 | 0.87 | 3.70 | 1.8 | 0.1826 | 0.1213 | 43.7 | 1.776 |
| 2 | 1.39 | 1.3283 | 0.1841 | 0.85 | 2.27 | 0.1 | 0.787 | 0.000 | 0.0 | 1.000 |
| 3 | 1.93 | 2.1949 | 0.0282 | 1.07 | 3.49 | 1.1 | 0.2926 | 0.018 | 9.7 | 1.1074 |
| **BMI overweight endometroid** | |  |  |  |  |  |  |  |  |  |
| study | estimate | zval | pval | ci.lb | ci.ub | Q | Qp | tau2 | I2 | H2 |
| 1 | 1.27 | 1.901 | 0.0573 | 0.99 | 1.64 | 3.8 | 0.4408 | 0.0000 | 0.0 | 1.000 |
| 2 | 1.24 | 1.4326 | 0.152 | 0.92 | 1.65 | 3.9 | 0.4204 | 0.0000 | 0.0 | 1.0000 |
| 3 | 1.24 | 1.4834 | 0.138 | 0.93 | 1.64 | 3.9 | 0.4214 | 0.0000 | 0.0 | 1.0000 |
| 4 | 1.25 | 1.6294 | 0.1032 | 0.96 | 1.65 | 3.9 | 0.4155 | 0.0000 | 0.0 | 1.0000 |
| 5 | 1.31 | 2.1001 | 0.0357 | 1.02 | 1.69 | 2.8 | 0.5916 | 0.0000 | 0.0 | 1.0000 |
| 6 | 1.22 | 1.5793 | 0.1143 | 0.95 | 1.55 | 1.2 | 0.8833 | 0.0000 | 0.0 | 1.0000 |
| **BMI endometroid obesity** | |  |  |  |  |  |  |  |  |  |
| study | estimate | zval | pval | ci.lb | ci.ub | Q | Qp | tau2 | I2 | H2 |

| 1 | 1.20 | 1.082 | 0.2792 | 0.8651 | 1.6523 | 0.5 | 0.7814 | 0.0000 | 0.0000 | 1.0000 |
| --- | --- | --- | --- | --- | --- | --- | --- | --- | --- | --- |
| 2 | 1.13 | 0.5516 | 0.5812 | 0.7307 | 1.7497 | 1.1 | 0.575 | 0.0000 | 0.0000 | 1.0000 |
| 3 | 1.09 | 0.436 | 0.6629 | 0.7424 | 1.5973 | 0.8 | 0.676 | 0.0000 | 0.0000 | 1.0000 |
| 4 | 1.21 | 1.0813 | 0.2796 | 0.8562 | 1.7118 | 0.8 | 0.6603 | 0.0000 | 0.0000 | 1.0000 |
| **BMI serous overweight** | |  |  |  |  |  |  |  |  |  |
| study | estimate | zval | pval | ci.lb | ci.ub | Q | Qp | tau2 | I2 | H2 |
| 1 | 1.04 | 0.4098 | 0.682 | 0.85 | 1.29 | 9.9 | 0.0792 | 0.0273 | 43.8233 | 1.8 |
| 2 | 1.18 | 1.1191 | 0.2631 | 0.88 | 1.59 | 13.9 | 0.016 | 0.0838 | 70.1429 | 3.3 |
| 3 | 1.14 | 0.7472 | 0.4549 | 0.81 | 1.60 | 12.9 | 0.0245 | 0.1104 | 66.3291 | 3.0 |
| 4 | 1.22 | 1.3038 | 0.1923 | 0.90 | 1.63 | 10.5 | 0.0615 | 0.0691 | 56.2034 | 2.3 |
| 5 | 1.20 | 1.1458 | 0.2519 | 0.88 | 1.64 | 13.5 | 0.0187 | 0.0886 | 68.1003 | 3.1 |
| 6 | 1.17 | 0.9985 | 0.3181 | 0.86 | 1.58 | 14.4 | 0.0131 | 0.0903 | 71.8446 | 3.6 |
| 7 | 1.04 | 0.365 | 0.7151 | 0.85 | 1.27 | 9.5 | 0.0902 | 0.0235 | 39.9448 | 1.7 |
| **BMI serous obesity** | |  |  |  |  |  |  |  |  |  |
| Study | estimate | zval | pval | ci.lb | ci.ub | Q | Qp | tau2 | I2 | H2 |
| 1 | 0.95 | -0.4416 | 0.6588 | 0.76 | 1.19 | 1.0 | 0.5937 | 0.0000 | 0.0 | 1.0000 |
| 2 | 1.02 | 0.0935 | 0.9255 | 0.73 | 1.41 | 0.8 | 0.6867 | 0.0000 | 0.0 | 1.0000 |
| 3 | 0.95 | -0.3826 | 0.702 | 0.74 | 1.22 | 1.0 | 0.5928 | 0.0000 | 0.0 | 1.0000 |
| 4 | 0.91 | -0.7799 | 0.4354 | 0.73 | 1.15 | 0. | 0.9859 | 0.0000 | 0.0 | 1.0000 |
| **BMI clear cell overweight** | |  |  |  |  |  |  |  |  |  |
| Study | estimate | zval | pval | ci.lb | ci.ub | Q | Qp | tau2 | I2 | H2 |
| 1 | 2.15 | 1.5333 | 0.1252 | 0.81 | 5.72 | 0.0 | 1.0000 | 0.0000 | 0.0 | 1.0000 |
| 2 | 1.50 | 1.2732 | 0.2029 | 0.80 | 2.80 | 0.0 | 1.0000 | 0.0000 | 0.0 | 1.0000 |
| **BMI clear cell obesity** | |  |  |  |  |  |  |  |  |  |
| Study | estimate | zval | pval | ci.lb | ci.ub | Q | Qp | tau2 | I2 | H2 |

| 1 | 2.26 | 3.5457 | 0.0004 | 1.44 | 3.55 | 0.3 | 0.8824 | 0.0000 | 0.0 | 1.000 |
| --- | --- | --- | --- | --- | --- | --- | --- | --- | --- | --- |
| 2 | 1.54 | 1.0771 | 0.2814 | 0.70 | 3.36 | 3.7 | 0.1577 | 0.2164 | 45.3 | 1.8295 |
| 3 | 1.59 | 1.3243 | 0.1854 | 0.80 | 3.14 | 4.0 | 0.1377 | 0.18 | 49.2 | 1.9697 |
| 4 | 1.70 | 1.3425 | 0.1794 | 0.78 | 3.71 | 4.5 | 0.1049 | 0.2688 | 56.8 | 2.3125 |
| **BMI overweight other** | |  |  |  |  |  |  |  |  |  |
| Study | estimate | zval | pval | ci.lb | ci.ub | Q | Qp | tau2 | I2 | H2 |
| 1 | 1.53 | 1.2804 | 0.2004 | 0.80 | 3.00 | 0.3 | 0.5709 | 0.0000 | 0.0 | 1.0000 |
| 2 | 0.91 | -0.258 | 0.7964 | 0.43 | 1.91 | 3.0 | 0.0846 | 0.1954 | 66.4 | 2.9743 |
| 3 | 0.98 | -0.0292 | 0.9767 | 0.31 | 3.11 | 2.4 | 0.1206 | 0.4574 | 58.5 | 2.4091 |
| **BMI obesity other** | |  |  |  |  |  |  |  |  |  |
| Study | estimate | zval | pval | ci.lb | ci.ub | Q | Qp | tau2 | I2 | H2 |
| 1 | 1.80 | 1.8205 | 0.0687 | 0.96 | 3.39 | 0.2 | 0.6937 | 0.0000 | 0.0 | 1.0000 |
| 2 | 1.55 | 3.3429 | 0.0008 | 1.20 | 2.01 | 0.1 | 0.7821 | 0.0000 | 0.0 | 1.0000 |
| 3 | 1.55 | 3.1479 | 0.0016 | 1.18 | 2.04 | 0.3 | 0.5782 | 0.0000 | 0.0 | 1.0000 |

**Supporting Table S8** Risk of bias assessment using ROBINS-I

| **Author, Year** | **con- founding** | **selection bias** | **information bias** | **reporting bias** | **Period of life** | **Ovarian cancer type** |
| --- | --- | --- | --- | --- | --- | --- |
| Nunez et al., 2017(1) | low | moderate | moderate | low | - | total |
| Bandera et al., 2016(2) | low | low | moderate | low | young adulthood, pre- & postmenopause | epithelial |
| Schildkraut et al., 2014(3) | low | low | moderate | low | - | epithelial |
| Su et al., 2012(4) | low | moderate | moderate | low | - | epithelial/ mucinous |
| Delort et al., 2009(5) | low | moderate | moderate | low | young adulthood, pre- & postmenopause | total |
| Moorman et al.,2009(6) | low | low | low | low | - | epithelial |
| Moorman et al.,2009(6) | low | low | low | low | - | epithelial |
| Olsen et al.,2008(7) | low | low | moderate | low | young adulthood, pre- & postmenopause | epithelial/ mucinous/ clear cell |
| Soegaard et al., 2007(8) | moderate | low | moderate | low | - | epithelial/ mucinous |
| Beehler et al., 2006(9) | low | moderate | moderate | low | pre- & postmenopause | epithelial |
| Rossing et al.,2006(10) | low | low | moderate | low | young adulthood | epithelial |
| Peterson et al. 2006(11) | low | low | moderate | low | young adulthood, pre- & postmenopause | epithelial |
| Greer et al,2005(12) | low | low | moderate | low | young adulthood | epithelial |
| Hoyo et al., 2005(13) | low | low | moderate | low | young adulthood | epithelial |
| Zhang et al., 2005(14) | low | moderate | moderate | low | young adulthood | epithelial |
| Riman et al., 2004(15) | low | low | moderate | low | - | epithelial/ mucinous/ clear cell |
| Pike et al., 2004(16) | low | low | moderate | low | - | total |
| Lubin et al., 2003(17) | low | low | moderate | low | young adulthood- | epithelial |
| Dal Maso et al., 2002(18) | low | moderate | moderate | low | - | epithelial |
| Kuper et al., 2002(19) | low | low | moderate | low | pre- & postmenopause | epithelial/ mucinous |
| Lukanova et al.,2002(20) | low | low | low | moderate | - | epithelial |
| Purdie et al., 2001(21) | low | low | moderate | low | pre- & postmenopause | epithelial/ mucinous/ clear cell |
| Hirose et al.,1999(22) | moderate | moderate | moderate | low | - | total |
| Ness et al.,2000(23) | low | low | moderate | low | - | epithelial |
| Mori et al., 1998(24) | moderate | moderate | moderate | low | - | epithelial |
| Farrow et al., 1989(25) | low | low | moderate | low | - | epithelial/ mucinous |
| Yang et al., 2012(26) | low | low | moderate | low | - | epithelial/ mucinous/ clear cell |
| Bjᴓrge et al., 2011(27) | low | low | low | low | - | epithelial |
| Brändstedt et al.,2011(28) | low | low | low | low | - | epithelial |
| Kotstopolus et al.,2010(29) | low | low | moderate | low | pre- & postmenopause | epithelial |
| Leitzmann et al.2009(30) | low | low | moderate | low | young adulthood | epithelial |
| Reeves et al., 2007(31) | low | low | moderate | low | - | total |
| Lundqvist et al., 2007(32) | low | low | moderate | low | - | total |
| Lundqvist et al., 2007(32) | low | low | moderate | low | - | total |
| Lacey et al., 2006(33) | low | moderate | low | low | - | total |
| Lukanova et al.,2006(34) | low | low | low | low | - | epithelial |
| Anderson et al.,2004(35) | low | low | moderate | low | young adulthood | epithelial |
| Kuriyama et al.,2004(36) | low | low | moderate | low | - | total |
| Niwa et al., 2004(37) | low | low | moderate | low | - | total |
| Engeland et al.,2003(38) | moderate | low | low | low | - | total |
| Schouten et al.,2003(39) | low | low | moderate | low | young adulthood | epithelial |
| Fairfield et al.,2002(40) | low | moderate | moderate | low | young adulthood, pre- & postmenopause | epithelial |

| **low** | **moderate** | **serious** | **critical** |
| --- | --- | --- | --- |

**Supporting Table S9**: Quality rating of the association between obesity and ovarian cancer for overall incident ovarian cancer and the main subgroup analyses using GRADE

| N studies | study design | risk of bias | Incon-sistency | indirectness | imprecision | other con- siderations | N controls | N obesity | RR (95% CI) | quality |
| --- | --- | --- | --- | --- | --- | --- | --- | --- | --- | --- |
| total incident ovarian cancer | | | | | | | | | | |
| 35 | observation | not serious | not serious | not serious | not serious | not serious | 4576 | 4031 | 1.19 (1.11-1.28) | ⨁⨁◯◯ low |
| ovarian cancer due to excess weight in young adulthood | | | | | | | | | | |
| 7 | observation | not serious | not serious | not serious | not serious | not serious | 361 | 476 | 1.39 (1.10-1.75) | ⨁⨁◯◯ low |
| ovarian cancer due to premenopausal excess weight | | | | | | | | | | |
| 9 | observation | not serious | not serious | not serious | not serious | not serious | 506 | 511 | 1.51 (1.21-1.88) | ⨁⨁◯◯ low |
| ovarian cancer due to postmenopausal excess weight | | | | | | | | | | |
| 9 | observation | not serious | not serious | not serious | not serious | not serious | 609 | 1360 | 1.03 (0.82-1.31) | ⨁⨁◯◯ low |
| mucinous ovarian cancer subtype | | | | | | | | | | |
| 5 | observation | not serious | not serious | not serious | not serious | not serious | 192 | 1633 | 1.44 (1.03-2.01) | ⨁⨁◯◯ low |
| clear cell ovarian cancer subtype | | | | | | | | | | |
| 4 | observation | not serious | not serious | not serious | not serious | not serious | 247 | 1633 | 1.82 (1.11-2.99) | ⨁⨁◯◯ low |

**Resource 1** Prisma Checklist 2020

| **Section and Topic** | **Item #** | **Checklist item** | **Location where item is reported** |
| --- | --- | --- | --- |
| **TITLE** | | |  |
| Title | 1 | Identify the report as a systematic review. | Page 1 |
| **ABSTRACT** | | |  |
| Abstract | 2 | See the PRISMA 2020 for Abstracts checklist. | Page 2 |
| **INTRODUCTION** | | |  |
| Rationale | 3 | Describe the rationale for the review in the context of existing knowledge. | Page 3 |
| Objectives | 4 | Provide an explicit statement of the objective(s) or question(s) the review addresses. | Page 3 |
| **METHODS** | | |  |
| Eligibility criteria | 5 | Specify the inclusion and exclusion criteria for the review and how studies were grouped for the syntheses. | Page 4 |
| Information sources | 6 | Specify all databases, registers, websites, organisations, reference lists and other sources searched or consulted to identify studies. Specify the date when each source was last searched or consulted. | Page 4 |
| Search strategy | 7 | Present the full search strategies for all databases, registers and websites, including any filters and limits used. | Page 4 |
| Selection process | 8 | Specify the methods used to decide whether a study met the inclusion criteria of the review, including how many reviewers screened each record and each report retrieved, whether they worked independently, and if applicable, details of automation tools used in the process. | Page 4 |
| Data collection process | 9 | Specify the methods used to collect data from reports, including how many reviewers collected data from each report, whether they worked independently, any processes for obtaining or confirming data from study investigators, and if applicable, details of automation tools used in the process. | Page 4 |
| Data items | 10a | List and define all outcomes for which data were sought. Specify whether all results that were compatible with each outcome domain in each study were sought (e.g. for all measures, time points, analyses), and if not, the methods used to decide which results to collect. | Page 4 |
|  | 10b | List and define all other variables for which data were sought (e.g. participant and intervention characteristics, funding sources). Describe any assumptions made about any missing or unclear information. | Page 4-5 |
| Study risk of bias assessment | 11 | Specify the methods used to assess risk of bias in the included studies, including details of the tool(s) used, how many reviewers assessed each study and whether they worked independently, and if applicable, details of automation tools used in the process. | Page 5 |
| Effect measures | 12 | Specify for each outcome the effect measure(s) (e.g. risk ratio, mean difference) used in the synthesis or presentation of results. | Page 4-5 |
| Synthesis methods | 13a | Describe the processes used to decide which studies were eligible for each synthesis (e.g. tabulating the study intervention characteristics and comparing against the planned groups for each synthesis (item #5)). | Page 5/Supporting Table S1 |
|  | 13b | Describe any methods required to prepare the data for presentation or synthesis, such as handling of missing summary statistics, or data conversions. | Page 5 |
|  | 13c | Describe any methods used to tabulate or visually display results of individual studies and syntheses. | Page 5 |
|  | 13d | Describe any methods used to synthesize results and provide a rationale for the choice(s). If meta-analysis was performed, describe the model(s), method(s) to identify the presence and extent of statistical heterogeneity, and software package(s) used. | Page 5 |
|  | 13e | Describe any methods used to explore possible causes of heterogeneity among study results (e.g. subgroup analysis, meta-regression). | Page 5 |
|  | 13f | Describe any sensitivity analyses conducted to assess robustness of the synthesized results. | Page 5 |
| Reporting bias assessment | 14 | Describe any methods used to assess risk of bias due to missing results in a synthesis (arising from reporting biases). | Page 5 |
| Certainty assessment | 15 | Describe any methods used to assess certainty (or confidence) in the body of evidence for an outcome. | Figure 2-3 |
| **RESULTS** | | |  |
| Study selection | 16a | Describe the results of the search and selection process, from the number of records identified in the search to the number of studies included in the review, ideally using a flow diagram. | Figure 1/Page 6 |
|  | 16b | Cite studies that might appear to meet the inclusion criteria, but which were excluded, and explain why they were excluded. | Page 6 |
| Study characteristics | 17 | Cite each included study and present its characteristics. | Supporting Table S1 |
| Risk of bias in studies | 18 | Present assessments of risk of bias for each included study. | Supporting Table S8/S9  Page 8-9 |
| Results of individual studies | 19 | For all outcomes, present, for each study: (a) summary statistics for each group (where appropriate) and (b) an effect estimate and its precision (e.g. confidence/credible interval), ideally using structured tables or plots. | Figures 2-3 |
| Results of syntheses | 20a | For each synthesis, briefly summarise the characteristics and risk of bias among contributing studies. | N/A |
|  | 20b | Present results of all statistical syntheses conducted. If meta-analysis was done, present for each the summary estimate and its precision (e.g. confidence/credible interval) and measures of statistical heterogeneity. If comparing groups, describe the direction of the effect. | Supporting Table S2 |
|  | 20c | Present results of all investigations of possible causes of heterogeneity among study results. | Supporting Table S2  Page 7/8 |
|  | 20d | Present results of all sensitivity analyses conducted to assess the robustness of the synthesized results. | Supporting Table S4-S7  Page 7/8 |
| Reporting biases | 21 | Present assessments of risk of bias due to missing results (arising from reporting biases) for each synthesis assessed. | Supporting Figure 4 |
| Certainty of evidence | 22 | Present assessments of certainty (or confidence) in the body of evidence for each outcome assessed. | Figures 2-4/Supporting Table S2 |
| **DISCUSSION** | | |  |
| Discussion | 23a | Provide a general interpretation of the results in the context of other evidence. | Page 9-11 |
|  | 23b | Discuss any limitations of the evidence included in the review. | Page 12 |
|  | 23c | Discuss any limitations of the review processes used. | N/A |
|  | 23d | Discuss implications of the results for practice, policy, and future research. | Page 12 |
| **OTHER INFORMATION** | | |  |
| Registration and protocol | 24a | Provide registration information for the review, including register name and registration number, or state that the review was not registered. | N/A |
|  | 24b | Indicate where the review protocol can be accessed, or state that a protocol was not prepared. | Page 4 |
|  | 24c | Describe and explain any amendments to information provided at registration or in the protocol. | N/A |
| Support | 25 | Describe sources of financial or non-financial support for the review, and the role of the funders or sponsors in the review. | Page 1 |
| Competing interests | 26 | Declare any competing interests of review authors. | Page 1 |
| Availability of data, code and other materials | 27 | Report which of the following are publicly available and where they can be found: template data collection forms; data extracted from included studies; data used for all analyses; analytic code; any other materials used in the review. | Resource 2/  Page 4 |

**Resource 2** Search Terms

Search Term Pubmed:

(obese[Title/Abstract] OR obesity[Title/Abstract] OR adiposity[Title/Abstract] OR body mass index[Title/Abstract] OR BMI[Title/Abstract] OR body weight[Title/Abstract] OR body size[title/abstract] OR anthropometry[MeSH Terms]) AND (ovarian cancer[Title/Abstract] OR ovarian tumor[Title/Abstract] OR ovarian neoplasms[Title/Abstract] OR ovarian carcinoma[title/abstract] OR ovary cancer[Title/Abstract] OR ovary neoplasms[MeSH Terms]) AND (risk[Title/Abstract] OR site- specific[Title/Abstract] OR incidence[Title/Abstract] OR hazard [title/abstract] OR prognostic factor[title/abstract]) NOT (editorial[Publication Type] OR comment[Publication Type] OR letter[Publication Type] OR guideline[Publication Type] OR news[Publication Type] OR review[Publication Type]) AND humans[MeSH Terms]

Search Term Web of Science:

(TI=(obesity OR obese OR BMI OR body mass index OR body size OR body weight OR adiposity OR anthropometry) OR TS=(obesity OR obese OR BMI OR body mass index OR body size OR body weight OR adiposity OR anthropometry)) AND (TI=(ovarian cancer OR ovarian tumor OR ovarian neoplasms OR ovarian carcinoma OR ovary cancer OR ovarian neoplasms) OR TS=(ovarian cancer OR ovarian tumor OR ovarian neoplasms OR ovarian carcinoma OR ovary cancer OR ovarian neoplasms)) AND (TI=(risk OR site-specific OR site-specific cancer incidence OR hazard OR prognostic factor OR incidence) OR TS=(risk OR site-specific OR site-specific cancer incidence OR hazard OR prognostic factor OR incidence)) AND SU=(Health Care Sciences & Services OR Oncology)AND LANGUAGE: (English OR German) AND DOCUMENT TYPES: (Article

Literature Cited

1. Nunez C, Bauman A, Egger S, Sitas F, Nair-Shalliker V. Obesity, physical activity and cancer risks: Results from the Cancer, Lifestyle and Evaluation of Risk Study (CLEAR). Cancer Epidemiol 2017; 47:56–63.

2. Bandera EV, Qin B, Moorman PG, Alberg AJ, Barnholtz-Sloan JS, Bondy M et al. Obesity, weight gain, and ovarian cancer risk in African American women. Int. J. Cancer 2016; 139(3):593–600.

3. Schildkraut JM, Alberg AJ, Bandera EV, Barnholtz-Sloan J, Bondy M, Cote ML et al. A multi-center population-based case–control study of ovarian cancer in African-American women: the African American Cancer Epidemiology Study (AACES). BMC Cancer 2014; 14(1):10.

4. Su D, Pasalich M, Binns CW, Lee AH. Is body size associated with ovarian cancer in southern Chinese women? Cancer Causes Control 2012; 23(12):1977–84.

5. Delort L, Kwiatkowski F, Chalabi N, Satih S, Bignon Y-J, Bernard-Gallon DJ. Central adiposity as a major risk factor of ovarian cancer. Anticancer Res 2009; 29(12):5229–34.

6. Moorman PG, Palmieri RT, Akushevich L, Berchuck A, Schildkraut JM. Ovarian cancer risk factors in African-American and white women. Am J Epidemiol 2009; 170(5):598–606.

7. Olsen CM, Nagle CM, Whiteman DC, Purdie DM, Green AC, Webb PM. Body size and risk of epithelial ovarian and related cancers: a population-based case-control study. Int J Cancer 2008; 123(2):450–6.

8. Soegaard M, Jensen A, Høgdall E, Christensen L, Høgdall C, Blaakaer J et al. Different risk factor profiles for mucinous and nonmucinous ovarian cancer: results from the Danish MALOVA study. Cancer Epidemiol Biomarkers Prev 2007; 16(6):1160–6.

9. Beehler GP, Sekhon M, Baker JA, Teter BE, McCann SE, Rodabaugh KJ et al. Risk of ovarian cancer associated with BMI varies by menopausal status. J Nutr 2006; 136(11):2881–6.

10. Rossing MA, Tang M-TC, Flagg EW, Weiss LK, Wicklund KG, Weiss NS. Body size and risk of epithelial ovarian cancer (United States). Cancer Causes Control 2006; 17(5):713–20.

11. Peterson NB, Trentham-Dietz A, Newcomb PA, Chen Z, Gebretsadik T, Hampton JM et al. Relation of anthropometric measurements to ovarian cancer risk in a population-based case-control study (United States). Cancer Causes Control 2006; 17(4):459–67.

12. Greer JB, Modugno F, Ness RB, Allen GO. Anthropometry and the risk of epithelial ovarian cancer. Cancer 2006; 106(10):2247–57.

13. Hoyo C, Berchuck A, Halabi S, Bentley RC, Moorman P, Calingaert B et al. Anthropometric measurements and epithelial ovarian cancer risk in African-American and White women. Cancer Causes Control 2005; 16(8):955–63.

14. Zhang M, Xie X, Holman CDJ. Body weight and body mass index and ovarian cancer risk: a case-control study in China. Gynecol Oncol 2005; 98(2):228–34.

15. Riman T, Dickman PW, Nilsson S, Nordlinder H, Magnusson CM, Persson IR. Some life-style factors and the risk of invasive epithelial ovarian cancer in Swedish women. Eur J Epidemiol 2004; 19(11):1011–9.

16. Pike MC, Pearce CL, Peters R, Cozen W, Wan P, Wu AH. Hormonal factors and the risk of invasive ovarian cancer: a population-based case-control study. Fertil Steril 2004; 82(1):186–95.

17. Lubin F, Chetrit A, Freedman LS, Alfandary E, Fishler Y, Nitzan H et al. Body mass index at age 18 years and during adult life and ovarian cancer risk. Am J Epidemiol 2003; 157(2):113–20.

18. Dal Maso L, Franceschi S, Negri E, Conti E, Montella M, Vaccarella S et al. Body size indices at different ages and epithelial ovarian cancer risk. European Journal of Cancer 2002; 38(13):1769–74.

19. Kuper H, Cramer DW, Titus-Ernstoff L. Risk of ovarian cancer in the United States in relation to anthropometric measures: does the association depend on menopausal status? Cancer Causes Control 2002; 13(5):455–63.

20. Lukanova A, Toniolo P, Lundin E, Micheli A, Akhmedkhanov A, Muti P et al. Body mass index in relation to ovarian cancer: a multi-centre nested case-control study. Int J Cancer 2002; 99(4):603–8.

21. Purdie DM, Bain CJ, Webb PM, Whiteman DC, Pirozzo S, Green AC. Body size and ovarian cancer: case-control study and systematic review (Australia). Cancer Causes Control 2001; 12(9):855–63.

22. Hirose K, Tajima K, Hamajima N, Kuroishi T, Kuzuya K, Miura S et al. Comparative case-referent study of risk factors among hormone-related female cancers in Japan. Jpn J Cancer Res 1999; 90(3):255–61.

23. Ness RB, Grisso JA, Cottreau C, Klapper J, Vergona R, Wheeler JE et al. Factors related to inflammation of the ovarian epithelium and risk of ovarian cancer. Epidemiology 2000; 11(2):111–7.

24. Mori M, Nishida T, Sugiyama T, Komai K, Yakushiji M, Fukuda K et al. Anthropometric and other risk factors for ovarian cancer in a case-control study. Jpn J Cancer Res 1998; 89(3):246–53.

25. Farrow DC, Weiss NS, Lyon JL, Daling JR. Association of obesity and ovarian cancer in a case-control study. Am J Epidemiol 1989; 129(6):1300–4.

26. Yang HP, Trabert B, Murphy MA, Sherman ME, Sampson JN, Brinton LA et al. Ovarian cancer risk factors by histologic subtypes in the NIH-AARP diet and health study. Int. J. Cancer 2012; 131(4):938–48.

27. Bjorge T, Lukanova A, Tretli S, Manjer J, Ulmer H, Stocks T et al. Metabolic risk factors and ovarian cancer in the Metabolic Syndrome and Cancer project. International Journal of Epidemiology 2011; 40(6):1667–77.

28. Brändstedt J, Nodin B, Manjer J, Jirström K. Anthropometric factors and ovarian cancer risk in the Malmö Diet and Cancer Study. Cancer Epidemiol 2011; 35(5):432–7.

29. Kotsopoulos J, Baer HJ, Tworoger SS. Anthropometric measures and risk of epithelial ovarian cancer: results from the nurses’ health study. Obesity (Silver Spring) 2010; 18(8):1625–31.

30. Leitzmann MF, Koebnick C, Danforth KN, Brinton LA, Moore SC, Hollenbeck AR et al. Body mass index and risk of ovarian cancer. Cancer 2009; 115(4):812–22.

31. Reeves GK, Pirie K, Beral V, Green J, Spencer E, Bull D. Cancer incidence and mortality in relation to body mass index in the Million Women Study: cohort study. BMJ 2007; 335(7630):1134.

32. Lundqvist E, Kaprio J, Verkasalo PK, Pukkala E, Koskenvuo M, Söderberg KC et al. Co-twin control and cohort analyses of body mass index and height in relation to breast, prostate, ovarian, corpus uteri, colon and rectal cancer among Swedish and Finnish twins. Int J Cancer 2007; 121(4):810–8.

33. Lacey JV, Leitzmann M, Brinton LA, Lubin JH, Sherman ME, Schatzkin A et al. Weight, height, and body mass index and risk for ovarian cancer in a cohort study. Ann Epidemiol 2006; 16(12):869–76.

34. Lukanova A, Björ O, Kaaks R, Lenner P, Lindahl B, Hallmans G et al. Body mass index and cancer: results from the Northern Sweden Health and Disease Cohort. Int J Cancer 2006; 118(2):458–66.

35. Anderson JP, Ross JA, Folsom AR. Anthropometric variables, physical activity, and incidence of ovarian cancer: The Iowa Women’s Health Study. Cancer 2004; 100(7):1515–21.

36. Kuriyama S, Tsubono Y, Hozawa A, Shimazu T, Suzuki Y, Koizumi Y et al. Obesity and risk of cancer in Japan. Int. J. Cancer 2005; 113(1):148–57.

37. Niwa Y, Yatsuya H, Tamakoshi K, Nishio K, Kondo T, Lin Y et al. Relationship between body mass index and the risk of ovarian cancer in the Japanese population: findings from the Japanese Collaborate Cohort (JACC) study. J Obstet Gynaecol Res 2005; 31(5):452–8.

38. Engeland A, Tretli S, Bjorge T. Height, body mass index, and ovarian cancer: a follow-up of 1.1 million Norwegian women. J Natl Cancer Inst 2003; 95(16):1244–8.

39. Schouten LJ, Goldbohm RA, van den Brandt PA. Height, weight, weight change, and ovarian cancer risk in the Netherlands cohort study on diet and cancer. Am J Epidemiol 2003; 157(5):424–33.

40. Fairfield K. Obesity, weight gain, and ovarian cancer. Obstetrics & Gynecology 2002; 100(2):288–96.
